# Theft of host transferrin receptor-1 by *Toxoplasma gondii* is required for infection

**DOI:** 10.1128/mbio.01248-26

**Published:** 2026-10-23

**Authors:** Stephen L. Denton, Alexa Mejia, Lindsay L. Nevarez, Miguel P. Soares, Barbara A. Fox, David J. Bzik, Jason P. Gigley

**Affiliations:** 1 Department of Molecular Biology, University of Wyoming 4416, Laramie, Wyoming, USA; 2 Instituto Gulbenkian de Ciência 705996 , Lisbon, Portugal; 3 Department of Microbiology and Immunology, Geisel School of Medicine at Dartmouth, Lebanon, New Hampshire, USA; Iowa State University College of Veterinary Medicine, Ames, Iowa, USA

**Keywords:** *Toxoplasma gondii*, transferrin receptor, host protein theft, apicomplexan parasites

## Abstract

Host nutrient acquisition is essential for apicomplexan parasite infection, yet mechanisms underpinning this are not defined. We discovered the genome of *Toxoplasma gondii* lacks conserved genes for iron transport and storage and these host cell proteins, including the transferrin receptor 1, transferrin, ferritin heart/heavy and liver/light chains, and clathrin light chain, are robustly taken up by tachyzoites. Acquisition of these host cell proteins was conserved across host cell species and parasite virulence types. Increased trafficking of host cell transferrin receptor 1 to endolysosomes boosted tachyzoite acquisition of host proteins and growth rate. Bradyzoites possessed little capacity to acquire host cell proteins, consistent with the cyst wall representing a barrier to host cell protein cargo. This study provides insight into essential functions associated with parasite theft of host iron acquisition and storage proteins.

**IMPORTANCE:**

Host nutrient theft is a dogmatic principle of parasitism. The paradigm that *Toxoplasma gondii* avoids association with host proteins, seemingly abstaining from them as a nutritional stream and for immune system avoidance, is changing. Many host proteins are enriched at the parasitophorous vacuole, but that barrier may be breached since exogenous proteins expressed by host cells transverse to *T. gondii* bodies. Our work presented here provides *in vivo* context to this new view because several host iron homeostatic proteins are acquired and retained within the parasite to support infection. These findings not only shed light on how *T. gondii* could acquire iron but also reveal new host-parasite interactions of theft and integration of host proteins by pathogens.

## INTRODUCTION

Upon infection, pathogens become confronted with a hostile microenvironment, whereby the host restricts access to vital nutrients and metabolites. In response, pathogens deploy virulence factors enabling the acquisition of nutrients, often in competition and to the detriment of the host. This general principle underlying host-pathogen interactions applies to intracellular eukaryotic parasites of the Apicomplexa phylum, which are responsible for global infectious diseases such as malaria (*Plasmodium* spp.) (1), cryptosporidiosis (*Cryptosporidium* spp.) (2), and toxoplasmosis (*Toxoplasma gondii*) (3).

*T. gondii* resides in a membrane-enclosed intracellular structure called the parasitophorous vacuole (PV). Previous studies have identified pathways for host-derived nutrient transit to the PV that support rapidly replicating *T. gondii* tachyzoites, including a PV pore (4), the Host Organelle-Sequestering Tubulostructure (5), and the Endosomal Sorting Complex Required for Transport (ESCRT) (6). Additional studies identified that the *T. gondii* micropore sequesters nutrients and proteins acquired from the host cell cytosol or Golgi (7) to support homeostasis of tachyzoite plasma membrane (PM) proteins (8). To date, no studies have addressed how *T. gondii* manipulates host iron homeostasis proteins to acquire iron.

The vital role of iron in redox-based biochemical pathways creates an aggressive competition for iron between host and microbes. Virulence factors sequestering host iron include siderophores that bind and facilitate iron uptake, as well as pathogen mimics of host cellular iron transport machinery (9–12). Counterstrategies by the host restrict pathogen access to iron and are referred to as nutritional immunity (13). This defense response can cause intracellular iron accumulation, requiring reprogramming of iron metabolism to prevent iron toxicity.

Apicomplexan parasites generally require iron for successful infection (14, 15). In particular, iron is used by *T. gondii* for iron-sulfur complex loading, heme biosynthesis, cytochrome cofactors, reduction/oxidation, ROS management, in the divergent respiratory complex, and life-stage conversion (16–25). Thus, *T. gondii* growth is halted by iron chelation (23, 26, 27). In enterocytes, parasite growth inhibition by host cytokine IFNγ is rescued by iron salt supplementation and by iron-loaded holo-transferrin (27). This implies that innate iron nutritional immunity may be a critical resistance mechanism to control *T. gondii* infection and may be counteracted by *T. gondii* mechanisms. Indeed, *T. gondii* upregulates iron transporter transferrin receptor (TfR) in fibroblasts (28, 29), expresses a vacuolar iron transporter (TgVIT) to protect the parasite from iron intoxication (26), and expresses a transporter (TgZFT) on the parasite PM to acquire iron from the PV lumen (30). *T. gondii* residence in the PV probably requires a unique set of iron-acquisition mechanisms to extract and manage iron from host cells. These, however, are not known.

Despite possessing intact gene pathways for heme biosynthesis, iron-sulfur complex loading, and intraparasite iron management, in this study, we show that *T. gondii* is devoid of genes encoding intact environmental iron extraction pathways. The sparse candidates identified do not encode a complete pathway and have low fitness costs when knocked out, suggesting that the parasite heavily relies on host iron machinery. In host cells, iron uptake is reportedly dependent on PM-localized TfR1 binding di-ferric transferrin (holo-Tf) and clathrin-mediated endocytosis (CME) of the complex (31). Endosomal acidification releases iron from holo-Tf, and iron is pumped into the host cell cytosol for use or for storage in the ferritin heavy chain (heart)/ferritin light chain (liver) (FHC/FLC) complex (32, 33). We reveal that *T. gondii* tachyzoites directly acquire host FHC, FLC, TfR1, and Tf. The acquisition of FHC, FLC, TfR1, and Tf occurs regardless of host species and parasite virulence, demonstrating a highly conserved and essential host-parasite interaction. Host TfR1 was inherited by newly formed daughter tachyzoites throughout endodyogeny. Theft of host TfR1 and Tf by *T. gondii* was required for parasite growth. While host clathrin light chain also was ingested by tachyzoites, increased endolysosomal trafficking of TfR1 significantly boosted parasite growth and TfR1 ingestion. Interestingly, host TfR1 was not significantly present in encysted bradyzoites of *T. gondii*. Altogether, this work uncovers novel parasite biology of sequestration of host proteins as integral components of the parasite body.

## MATERIALS AND METHODS

Additional experimental details, including expanded protocols, buffer recipes, antibodies and reagents (Table S1), and primers (Table S2), are provided as supplemental material.

### Tissue culture and animal maintenance

MRC5 human lung fibroblasts, Chinese hamster ovary (CHO) and CHO-derived cell lines, and murine ear fibroblasts (MFs) were used in this study. CHO-derived TRVb cells were kindly gifted by Dr. Caroline A. Enns, Oregon Health & Science University. TRVb-derived hTfR, mTfR, and mTfR-R654A complemented cells were kindly gifted by Dr. Paul J. Schmidt, Boston Children’s Hospital and Harvard School of Medicine. MFs were isolated from C57BL/6J mice using established protocols (34).

Type I strain RH and Type II strain Prugniaud parasites were maintained in MRC5 fibroblasts. Type II strain ME49 cysts were maintained in CBA/J mice.

C57BL/6J and Ubiquitin-CreER^T2^ mice were purchased from Jackson Laboratories. TfR1^Flx^ mice were kindly gifted by Dr. Mark Andrews (Harvard University). Ubiquitin-CreER^T2^ was crossed with TfR1^Flx^ to generate conditional TfR1 knockout mice (cTfR1KO).

### *T. gondii* cyst isolation and reactivation

Type II strain ME49 cysts were isolated from chronically infected mice via differential centrifugation as previously described (35), and bradyzoites were reactivated by inoculation of MRC5 fibroblasts. Parasite reactivation was assessed 24–36 hours after *in vitro* inoculation.

### Immunofluorescence assay

Confluent fibroblasts grown on coverslips were inoculated with 1 × 10^3^ *T. gondii* cells for 24 hours. Infected coverslips were fixed in 4% paraformaldehyde for 10 minutes at ambient temperature. Unless otherwise indicated, cells were permeabilized with Triton X-100 and blocked with FBS (PHEMBlock). Primary and secondary antibody staining occurred individually overnight in PHEMBlock at 4°C. Nuclei were stained with 2 µM DAPI in PBS.

Differential permeabilization was performed with Triton X-100, saponin, or digitonin in 3% FBS PHEM. Antibodies were diluted in 3% FBS PHEM without a permeabilization agent for otherwise identical immunostaining.

ME49-chronically infected C57BL/6 mice were perfused with 4% paraformaldehyde, and brains were sectioned on a freezing microtome, as described (36, 37). Fixed brain sections were blocked for 1 hour, then concurrently stained with *Dolichos biflorus* agglutinin (DBA)-FITC and anti-TfR1 overnight at 4°C, and a secondary stain occurred for 8 hours at 4°C. Slices were incubated with 2 µM DAPI and mounted on a microscope slide.

All slides were imaged on an Olympus IX81 confocal microscope with Metamorph v7.8.6.0 software and analyzed with ImageJ software v1.53f51.

### Parasite burdens

Fibroblast lines were inoculated with 100 *T. gondii* tachyzoites for 6 days. Cells were scraped and pelleted by centrifugation for genomic DNA extraction (DNeasy Blood & Tissue Kit, QIAGEN) and *T. gondii* genome quantification by quantitative real-time PCR targeting *T. gondii* unique gene B1 (38) using SSO Advanced SYBR Master Mix (Bio-Rad). Samples were compared to a standard curve of known *T. gondii* cell numbers.

### Quantitative reverse transcriptase PCR

RNA was isolated from cell pellets using TRI reagent (Sigma-Aldrich) and resuspended in DEPC-treated water supplemented with RNaseOUT (Invitrogen). Residual DNA was removed by the RNA Cleanup protocol of the RNeasy Kit (QIAGEN). cDNA was generated from 1 µg of RNA with Superscript IV Reverse Transcriptase (Invitrogen). Relative abundance between groups was estimated by relative TfR1 expression following PCR for mouse *Actin* (39) and *TfR1* (40) by the ∆∆Ct method.

### Western blot

Uninfected fibroblast lines or fibroblast lines infected with *T. gondii* at an MOI of 0.1 for 2 days were collected by scraping. Parasites were syringe-released and purified using a 27 G needle and filtration through a 3 µm membrane. Cells or purified parasites were lysed, and insoluble material was removed by centrifugation. Proteins were quantified by Bradford assay, normalized, and denatured by boiling in SDS loading buffer. Lysates (30 µg) were loaded in a 10% SDS-PAGE gel and transferred to PVDF. Membranes were blocked and blotted in 5% (wt/vol) non-fat milk in 0.1% Tween-20 in PBS. Blots were developed in ECL, and the chemiluminescence was imaged on an Invitrogen iBright. Uncropped images are provided in Fig. S1.

### Mass spectrometry

RH tachyzoites were inoculated into MRC5 fibroblasts as above. Following parasite destruction of the host cell monolayer, the parasites were purified in 3 µm pore filtration. Tachyzoites were washed three times with PBS and pelleted by centrifugation. Frozen pellets were sent to the University of Arkansas for Medical Sciences IDeA National Resource for Quantitative Proteomics facility for analysis. Reduced, alkylated, and chloroform/methanol purified proteins were digested with trypsin. Tryptic peptides were separated by a reverse-phase UHPLC and trapped (PepMap Neo). Eluate (Vanquish Neo UHPLC Nano System) was ionized by electrospray for mass spectrometric analysis on an Orbitrap Astral mass spectrometer. Data were analyzed using Spectronaut (Biognosys v20.1) against the UniProt *Homo sapiens* database (proteome ID: UP000005640, taxon ID: 9606, second version of 2025) and the *Toxoplasma gondii* database (proteome ID: UP000001529, taxon ID: 508771, second version of 2025) for peptide identification.

### Flow cytometry

Syringe-released purified tachyzoites or bradyzoites released from cysts were stained for viability with Live/Dead Aqua prior to 4% paraformaldehyde fixation. Parasites were sequentially stained with primary and secondary antibodies and analyzed on a Guava EasyCyte 12HT Flow Cytometer and with FlowJo v9.7.7 software.

### *In silico* identification of potential *T. gondii* iron-related genes

Iron-related proteins were manually selected and cataloged for query by literature review. Reference gene FASTA sequences (NCBI Protein Database) were queried for homology in the *Toxoplasma gondii* ME49 genome (ToxoDB Toxoplasma Bioinformatics Resource, ToxoDB.org) and the *Plasmodium falciparum* 3D7 genome (PlasmoDB Plasmodium Bioinformatics Resource, PlasmoDB.org), or the NCBI Taxon ID of other apicomplexans by pBLAST. Potential candidates were limited to those with bit scores greater than 50 and *E* values less than 1.00 × 10^−5^. Identified *T. gondii* genes were cross-referenced to CRISPR fitness scoring (41). All protein sequence alignments were performed by ClustalW analysis with a gap penalty of 10.0 in MacVector v17.0.10 software.

### Quantification and statistical analysis

Data were collected and organized in Microsoft Excel v16.54. GraphPad Prism v9 was used for statistics and graph generation. Statistical tests were Student’s *t*-test between two groups, ordinary one-way ANOVA between multiple groups, and two-way ANOVA between two groups with multiple conditions. Relevant statistical values are provided in figures and figure legends, including sample size (*n*), *P* values (*P*), *t* values (*t*), *F* values (*F*), and degrees of freedom (df). Figures were assembled in Adobe Illustrator 2021 and references in EndNote X8.

## RESULTS

### *Toxoplasma gondii* lacks conserved iron storage and transport machinery critical for iron homeostasis

**Fig 1:**
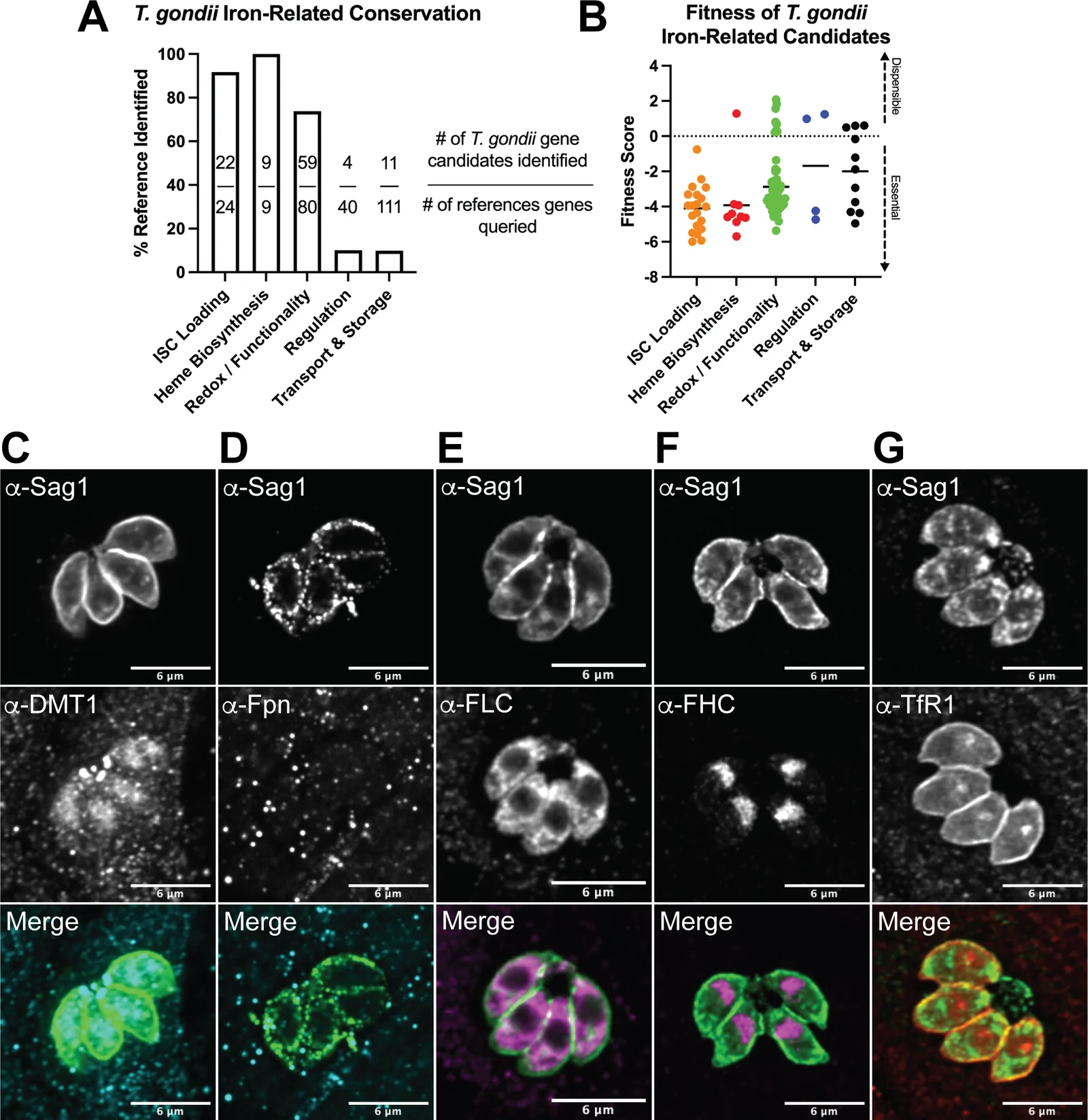
*T. gondii* lacks iron transport or storage genes, but host transferrin receptor and ferritin localize to the parasite. (**A and B**) Known iron-related proteins were categorized according to function in categories as follows: (i) iron-sulfur complex (ISC) loading, (ii) heme biosynthesis, (iii) reduction/oxidation production or management (redox/functionality), (iv) regulation by translation or transcription, or (v) storage and transport of iron. Protein sequence data were used as a query to probe the ME49 *T. gondii* genome for homology. (**A**) Category fulfillment as a function of the number of *T. gondii* genes identified out of the number of proteins queried. (**B**) Fitness metadata score of identified genes. (**C–G**) Twenty-four-hour post-infection RH strain *T. gondii* confocal IFA staining for host category 5 proteins (**C**) divalent metal transporter 1, (**D**) ferroportin, (**E**) ferritin (light chain), (**F**) ferritin (heavy chain), and (**G**) transferrin receptor 1.

To identify putative evolutionarily conserved iron regulatory genes in *T. gondii*, reference genes across genera were organized into categories and blasted against the genome of *T. gondii*. Categories included (i) iron-sulfur (Fe-S) loading complex, (ii) heme biosynthesis, (iii) redox functionality, (iv) transcription and translation, and (v) iron storage and transport (Table S3, summarized in Fig. 1A and B). The *T. gondii* genome contains complete homologs of Fe-S complex loading pathways, including members of the ISC pathway of bacteria, the Suf pathway of photosynthetic organisms, and the Nif pathway of eukaryotes (21, 42), as well as the heme biosynthesis pathway (17). In addition, as expected from previous studies (43–46), redox functionality was represented in the *T. gondii* genome, possessing several canonical iron-dependent functional proteins, such as superoxide dismutase and catalase, aconitase, and cytochromes C and P450, and cognate cytochrome oxidase C. The analysis revealed that *T. gondii* possesses few iron-dependent regulatory genes. There is a single copy of aconitase, which functionally acts as an iron regulatory protein 1 (IRP1, TGME49_226730) (25), and two Myb-domain containing proteins, one of which is BFD-1, the master regulator of differentiation (47).

Our analysis revealed only 10.6% homology to queried iron storage and transport proteins. No identified genes encoded an intact extracellular iron extraction pathway (Table 1). Four putative parasite-specific intracellular iron transporters were identified, including a transporter with homology to human NRAMP1/DMT1 and *Escherichia coli* MntH (TGME49_267270) (48, 49). Other cellular iron transporters were vacuolar iron transporter (VIT) (TGME49_266800) (26), ZIP (Zrt-Irt-like protein)-domain containing protein ZRT (TGME49_261720) (30), and ZIPCO (TGME49_225530) (50). The remaining identified candidates possess homology to disparate siderophore anabolism enzymes and transporters. In all, current understanding and this bioinformatic analysis highlight extensive regulation and utilization of iron by *T. gondii* but do not address how host cell iron homeostasis machinery contributes to parasite infection.

**TABLE 1:** Genomic conservation of putative *T. gondii* proteins that may extract iron from the host environment and detection of candidates within the *T. gondii* proteome^*a*^

Protein	Reference organism	NCBI accession no.	TG target ID	No. of peptides in TG lysate	UniProt accession no.	Table S3 reference
DMT1	*M. musculus*	NP_032758.2	TGME49_267270	14	S8EVW0	(1, 2)
NRAMP1	*M. musculus*	NP_038640.2				(3)
MntH	*E. coli*	NP_416893.1				(6)
VIT	*T. gondii*	TGME49_266800	TGME49_266800	12	B0Q1V7	(4)
YbtP	Enterobacteria	WP_001327262.1	TGME49_269000	0	S8EXU2	(5)
FecE	*Yersinia*	AAA97183.1				(10)
YbtQ	*E. coli*	WP_012131848.1	TGME49_273360	25	B6KDL5	(5)
YfeB	*Yersinia*	WP_002211843.1				(6)
EntA	*E. coli*	NP_415128.1	TGME49_217740	10	B6KU18	(7, 8)
PchD	*Pseudomonas aeruginosa*	CAA57966.1	TGME49_232580	0	B6KJV4	(9)
Irp9	Enterobacteria	VTP73725.1	TGME49_202920	0	S8F631	(11)
YbtS	Enterobacteria	WP_012132137.1				(12)
EntC	*E. coli*	NP_415125.1				(16)
PchA	*P. aeruginosa*	CAA57969.1				(14)
FhuC	*E. coli*	CAD6022026.1	TGME49_232580	0	A0A125YTI4	(13)
YbtE	Enterobacteria	WP_001088835.1	TGME49_266640	0	A0A125YT21	(12)
ZRT	*T. gondii*	TGME49_261720	TGME49_261720	12	B9PKE2	(15)
ZIPCO	*Plasmodium berghei*	PBANKA_0506500	TGME49_225530	0	S8EUF8	(15)
Ceruloplasmin	*H. sapiens*	ACB21047.1	No candidates	0	A5PL27	(26)
Ferroportin	*M. musculus*	AAF80987.1	No candidates	0	Q9NP59	(38, 39)
Ferritin Heavy Chain	*H. sapiens*	NP_002023.2	No candidates	8	P02794	(49)
Ferritin Light Chain	*H. sapiens*	NP_000137.2	No candidates	6	P02792	(49)
Hepcidin	*H. sapiens*	AAH20612.1	No candidates	0	P81172	(53)
HFE	*H. sapiens*	AAB82083.1	No candidates	2	Q30201	(54)
Lcn2	*H. sapiens*	XP_047279332.1	No candidates	0	P80188	(119)
TfR2	*H. sapiens*	NP_003218.2	No candidates	1	Q9UP52	(67)
TfRC	*H. sapiens*	AAA61153.1	No candidates	29	P02786	(68)
Transferrin	*M. musculus*	NP_598738.1	No candidates	0	P02787	(68)
ZIP8	*Rattus norvegicus*	AAH89844.1	No candidates	0	Q9C0K1	(117)
ZIP14	*M. musculus*	AAH21530.1	No candidates	4	Q15043	(118)

^*a*^ Alternating shading indicates one or more query proteins that returned the same *T. gondii* potential candidate.

### Host iron storage and transport machinery are incorporated by *T. gondii* tachyzoite bodies within the PV

Predominant host iron homeostasis proteins include DMT1 (import), Fpn (export), FHC/FLC (storage), and Tf/TfR1 (endocytosis) (32, 51–53). Since *T. gondii* lacks putative orthologs of host iron homeostasis machinery, except DMT1, we investigated the host cell localization of DMT1, Fpn, FHC, FLC, and TfR1 and the potential association of these proteins with tachyzoites residing in the PV. Intracellular *T. gondii*, identified by Sag1 immunofluorescence assay (IFA) (marking the tachyzoite PM), demonstrated bright DMT1 puncta at the apical end of the parasite (Fig. 1C). However, given the high homology of TGME49_267270 to host DMT1, this result was not explored further. Host Fpn exhibited no specific association with intracellular parasites and no apparent change of localization in infected cells (Fig. 1D).

**Fig 2:**
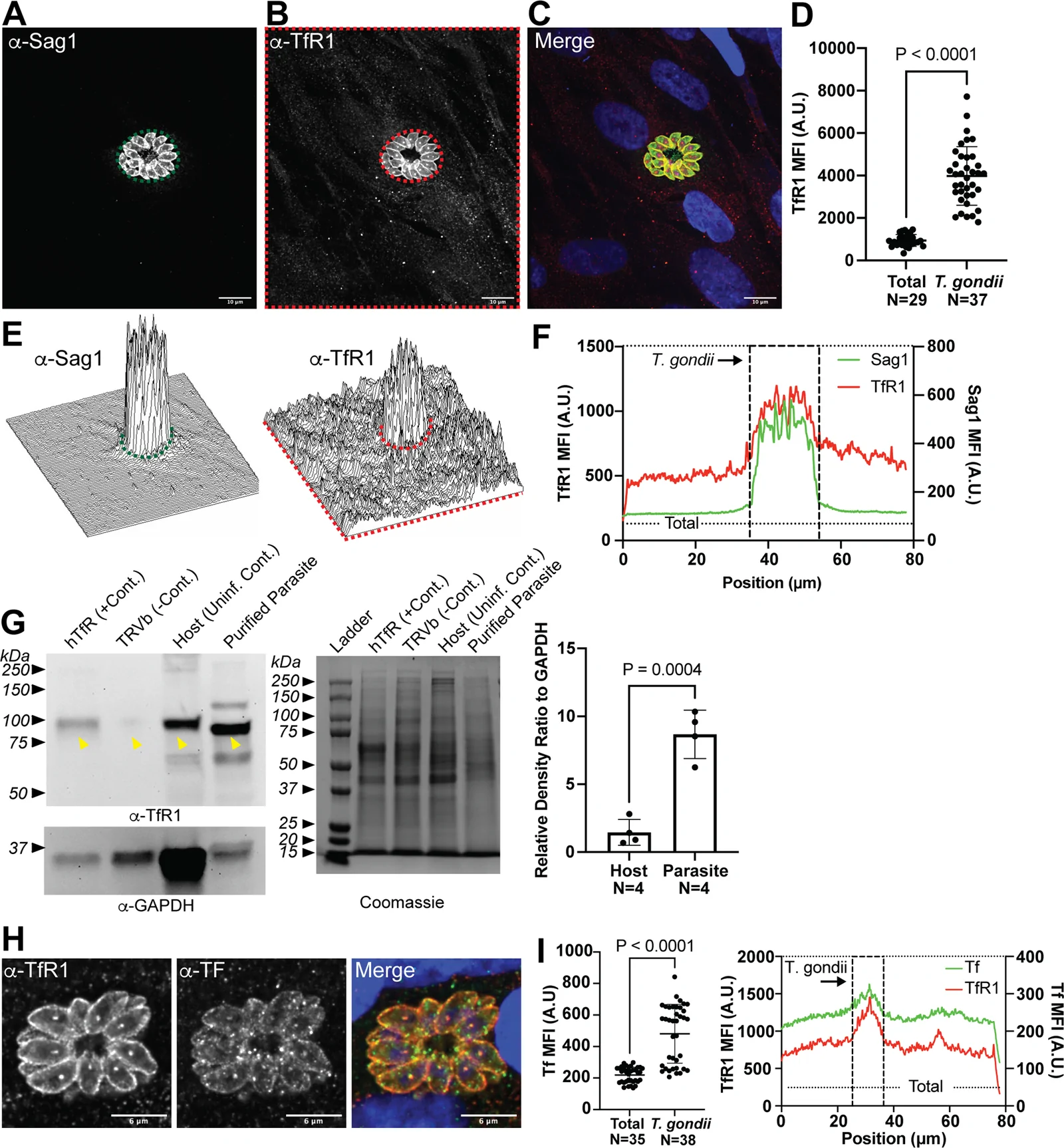
Host transferrin receptor 1 and cognate ligand transferrin are acquired by *T. gondii*. (**A–F**) Twenty-four-hour post-infection RH strain *T. gondii* and surrounding uninfected cell confocal IFA staining for host transferrin receptor 1 at low magnification. (**A**) Representative image of anti-Sag1 fluorescence to demonstrate parasite location. The parasite boundary is restricted to the area digitally denoted by the dashed green line. (**B**) Representative image of anti-TfR1 (JF0956) fluorescence. Boundary distinctions of parasite area (*T. gondii*), as determined by anti-Sag1 fluorescence, and the whole image (total) are denoted in dashed red lines. (**C**) Representative image of digitally merged Sag1 (green) and TfR1 (red) and DAPI (blue) fluorescence. (**D**) Comparison of relative mean fluorescence intensity (MFI) of TfR1 corresponding to the total image area (total) and the parasite-restricted area (*T. gondii*). The graph depicts centralized means and error bars representing the standard deviation of data pooled from four technical repeats and analyzed by unpaired Student’s *t*-test (*t* = 11.70, df = 64). (**E**) Representative surface plots displaying pixel intensity of Sag1 (left) and TfR1 (right) as further demonstration of boundary establishment. (**F**) Representative colocalization as overlayed distribution plots of y-slice MFI of Sag1 (green) and TfR1 (red). (**G**) Cell lysates (30 µg) were probed in Western blot for TfR1 (H68.4) (left). Positive control hTfR cell lysates are TfR1-deficient cells reconstituted with the human TfR1 gene. Negative control TRVb cells are the TfR1-deficient cell line. The uninfected control is the host cell lysate from uninfected MRC5 cells. Purified parasites are RH strain *T. gondii* mechanically lysed from host cells and filter purified. Coomassie stain of gel provided as an additional loading control (middle). Yellow arrows indicate the area tested for densitometry (right). The graphical data depict the centralized mean, and error bars represent the standard deviation of TfR1:GAPDH densitometry from four technical repeats, analyzed by unpaired Student’s *t*-test (*t* = 7.143, df = 6). Unaltered Western blot images and additional replicates are shown in the supplemental material. (**H**) High-magnification representative images of parasite confocal IFA staining for host TfR1 and Tf. (**I**) Representative distribution plot of TfR1 and Tf y-slice MFI; quantitation of parasite-associated Tf is represented by the centralized mean and error bars representing standard deviation of data pooled from three technical repeats and analyzed by Student’s *t*-test (*t* = 8.028, df = 71).

**Fig 3:**
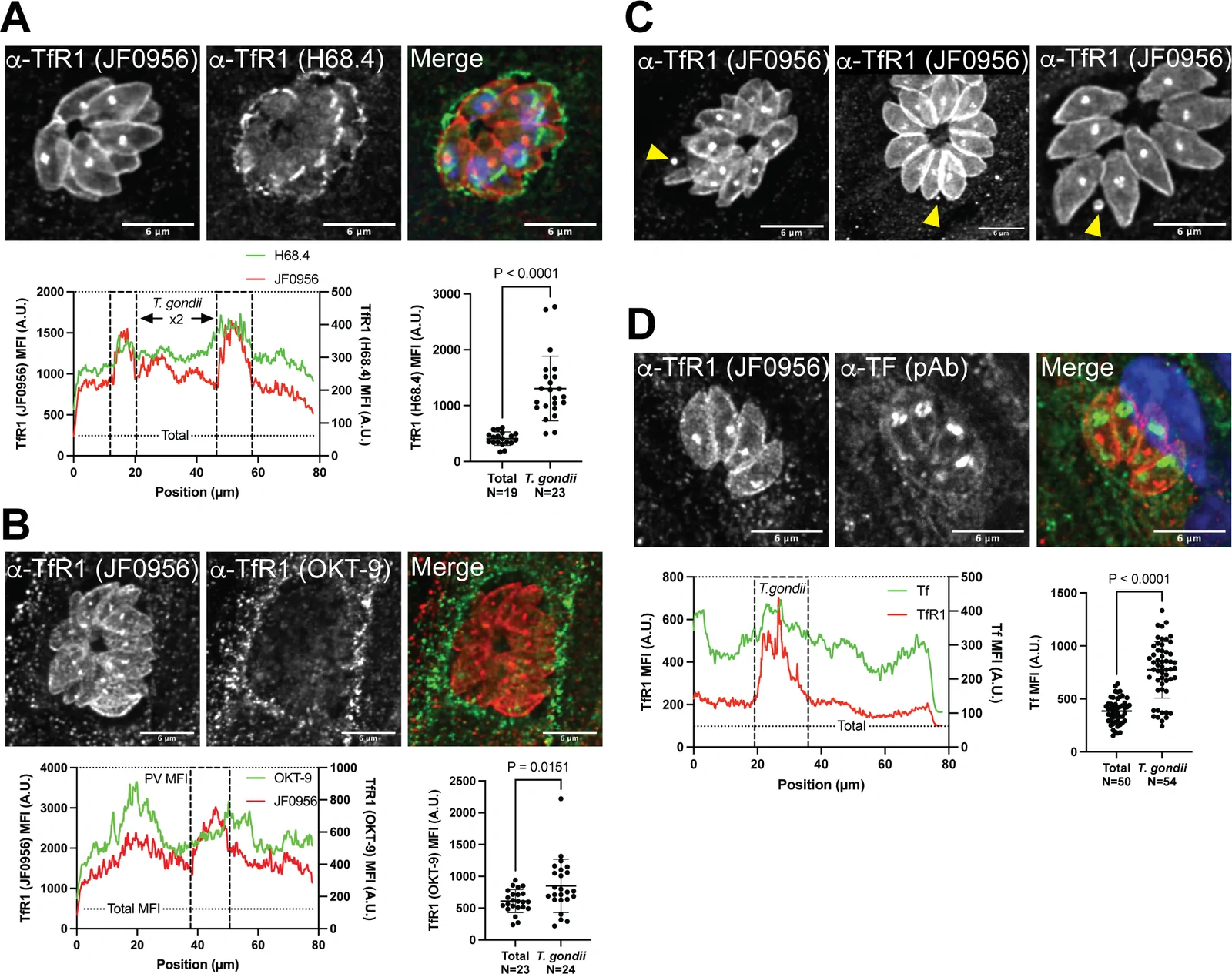
Localization of TfR1 and Tf to parasite niche is independent of antibody clonality. RH strain *T. gondii* 24-hour infections of MRC5 cells were stained for confocal IFA of host TfR1 and Tf. (**A**) Representative IFA and quantitation of parasite-associated TfR1 comparing JF0956 and H68.4 clones. Data pooled from two technical repeats and analyzed by unpaired Student’s *t*-test (*t* = 6.648, df = 40). (**B**) Representative IFA and quantitation of parasite-associated TfR1 comparing JF0956 and OKT-9 clones. Data pooled from two technical repeats and analyzed by unpaired Student’s *t*-test (*t* = 2.526, df = 45). (**C**) Chance observations of parasite-adjacent TfR1 (JF0956) puncta. (**D**) Representative IFA and quantitation of parasite-associated TfR1 (JF0956) and Tf (polyclonal, biotin-conjugated). Data pooled from two technical repeats and analyzed by unpaired Student’s *t*-test (*t* = 9.462, df = 102).

In contrast, we found an intense localization of host FHC, FLC, and TfR1 staining with intracellular *T. gondii* (Fig. 1E through G). There was significantly increased FHC and FLC mean fluorescence intensity (MFI) at the parasite location compared to surrounding host cells. FLC was detected throughout the *T. gondii* tachyzoite cytoplasm, whereas FHC staining appeared centrally localized (Fig. 1E and F). Host TfR1 outlined the periphery of the parasite PM and/or inner membrane complex (IMC) with a singular puncta of TfR1 fluorescence (Fig. 1G and Fig. 2A through C). Occasionally, TfR1 localized outside individual parasites but nearby, possibly within the PV lumen (Fig. 3C). Notably, the MFI of TfR1 of intracellular *T. gondii* was significantly higher than the surrounding host cells (Fig. 2D through F), and Z-stack imaging showed that it was not due to superimposition (Movie S1). Additional monoclonal antibodies against human TfR1 yielded similar localization to parasites (Fig. 3A and B), and Western blots also confirmed the presence of intact TfR1 in isolated parasites (Fig. 2G). However, the molecular weight of parasite-associated TfR1 was slightly downshifted from the host transferrin receptor (~97 kDa), and a new, higher MW band was apparent in parasite samples. Transferrin (Tf), the ligand for TfR1, also localized to intracellular *T. gondii* and had a higher MFI at this location than surrounding host cells (Fig. 2H and I and Fig. 3D; Movie S2).

**Fig 4:**
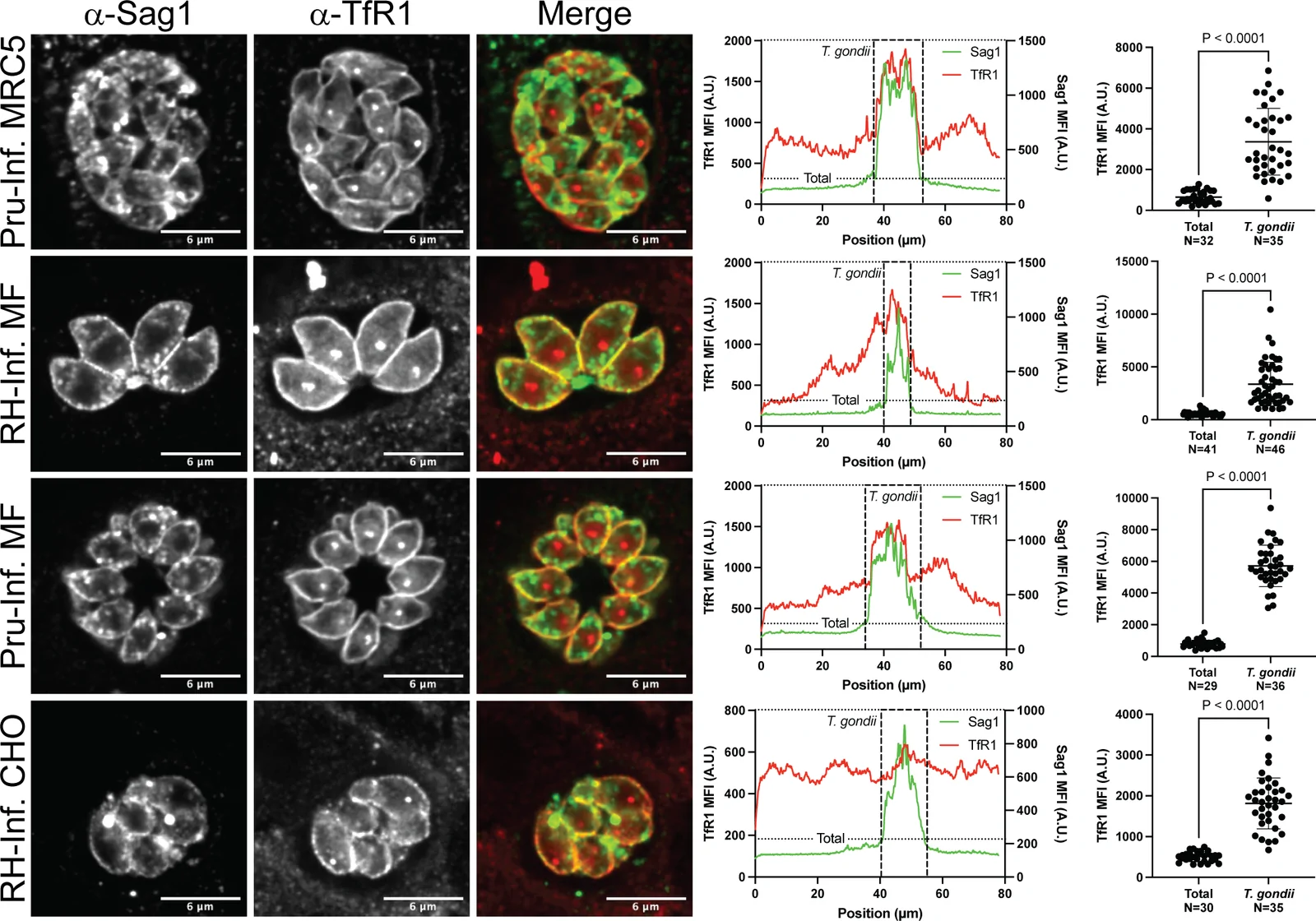
Transferrin receptor 1 theft occurs irrespective of virulence type or host cell genus. Virulence type I RH strain or type II Prugniaud (Pru) strain *T. gondii* inoculated into MRC5, MF, or CHO host cells was assayed for confocal TfR1 IFA. First (top) row: representative images, distribution plot, and quantitation of parasite-associated TfR1 of Pru-strain *T. gondii* infecting human (MRC5) cells. Second row: representative images, distribution plot, and quantitation of parasite-associated TfR1 of RH strain *T. gondii* infecting murine ear fibroblast (MF) cells. Third row: representative images, distribution plot, and quantitation of parasite-associated TfR1 of Pru-strain *T. gondii* infecting MF cells. Fourth (bottom) row: representative images, distribution plot, and quantitation of parasite-associated TfR1 of RH strain *T. gondii* infecting hamster CHO cells. Graphs of centralized mean and standard deviation in error bars depict data pooled from two independent repeats and analyzed by unpaired Student’s *t*-test (first row, *t* = 9.223, df = 65; second row, *t* = 8.543, df = 85; third row, *t* = 19.92, df = 63; fourth row, *t* = 11.25, df = 63).

**Fig 5:**
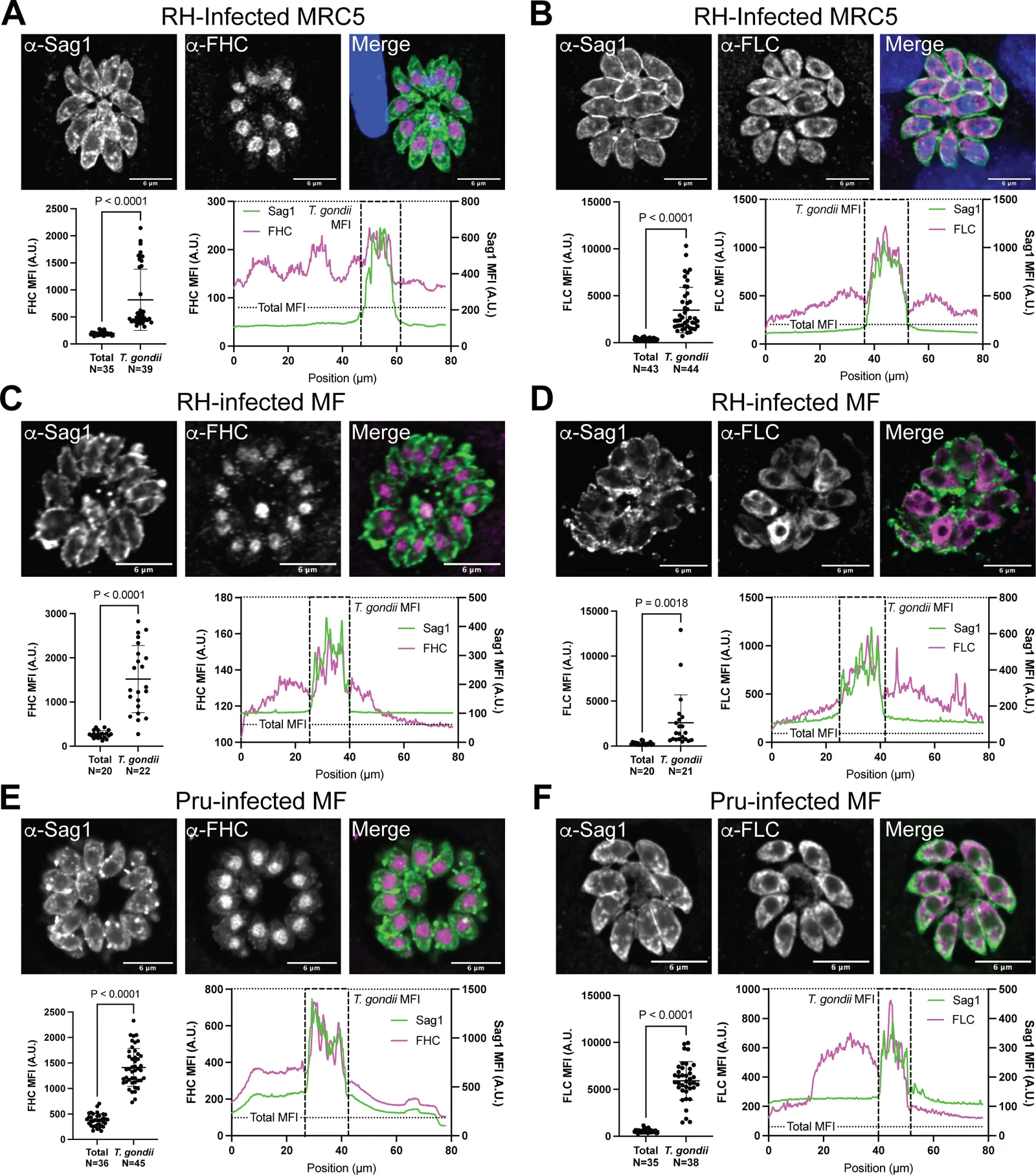
Theft of ferritin heavy and light chains occurs in multiple host cell species and by multiple parasite virulence types. Shown are representative images, distribution plots, and quantitation of parasite-associated (**A**) FHC of RH strain *T. gondii* infecting human (MRC5) cells. Data pooled from four technical repeats and analyzed by unpaired Student’s *t*-test (*t* = 6.485, df = 72). (**B**) FLC of RH strain *T. gondii* infecting human (MRC5) cells. Data pooled from four technical repeats and analyzed by unpaired Student’s *t*-test (*t* = 8.387, df = 85). (**C**) FHC of RH strain *T. gondii* infecting mouse (MF) cells. Data pooled from two technical repeats and analyzed by unpaired Student’s *t*-test (t = 7.350, df = 40). (**D**) FLC of RH strain *T. gondii* infecting mouse (MF) cells. Data pooled from two technical repeats and analyzed by unpaired Student’s *t*-test (t = 3.346, df = 39). (**E**) FHC of Pru-strain *T. gondii* infecting mouse (MF) cells. Data pooled from two technical repeats and analyzed by unpaired Student’s *t*-test (*t* = 15.95, df = 79). (**F**) FLC of Pru-strain *T. gondii* infecting mouse (MF) cells. Data pooled from two technical repeats and analyzed with unpaired Student’s *t*-test (*t* = 15.32, df = 71).

We tested if redistribution of host iron homeostatic proteins toward intracellular *T. gondii* was conserved across host species and parasite virulence. As revealed by IFA, parasite association of host FHC, FLC, and TfR1 was conserved, yielding similar morphologies in MFs and CHO cells compared to human fibroblasts (Fig. 4 and Fig. 5) and regardless of parasite virulence comparing highly virulent type I (RH) with avirulent type II (Pru) parasites.

**Fig 6:**
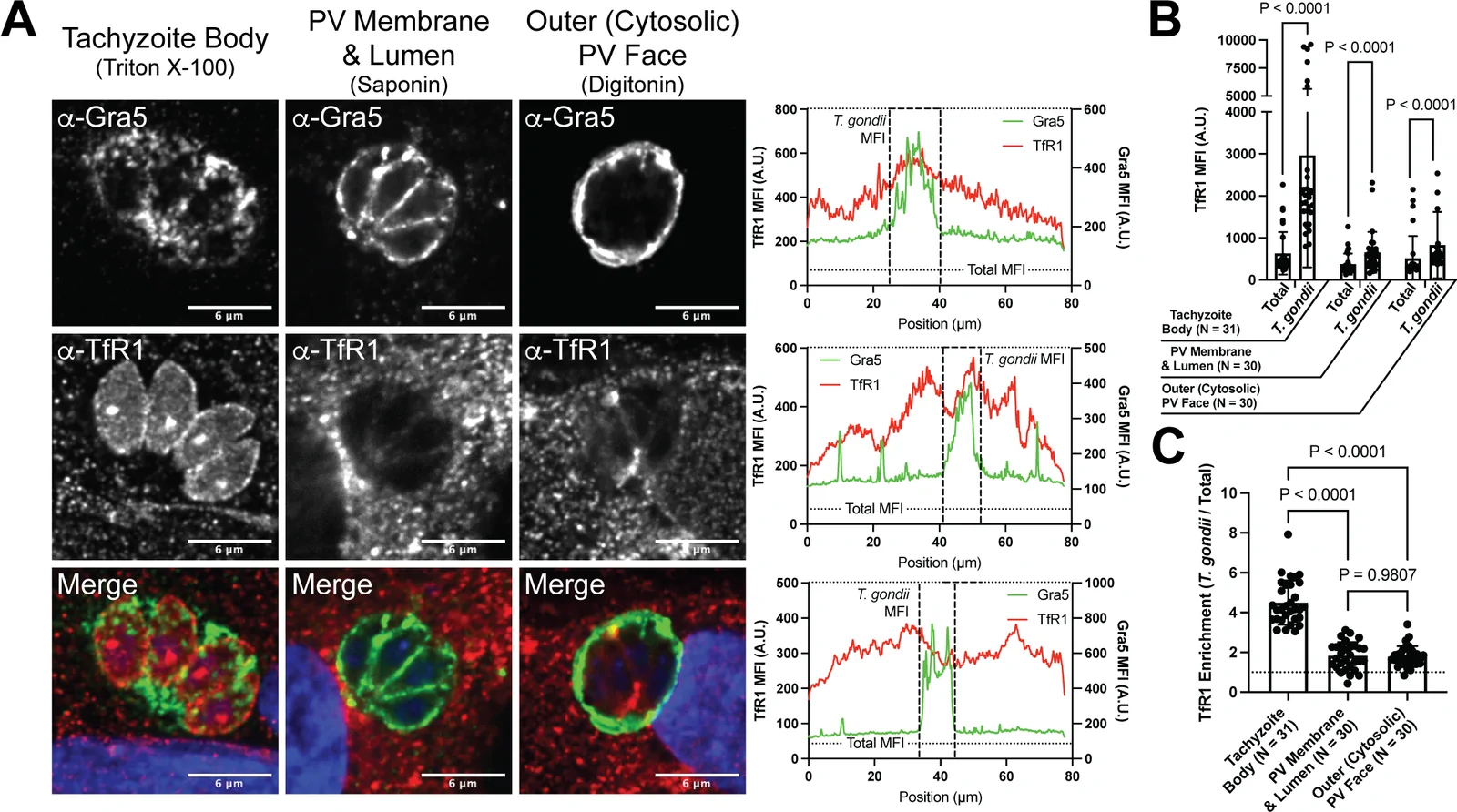
Host TfR1 is deposited on parasite cell bodies within the parasitophorous vacuole. RH strain *T. gondii* 24-hour infections of MRC5 cells were differentially permeabilized to differentially reveal subcellular compartments (digitonin, outer PV leaflet; saponin, PV membrane and lumen; Triton X-100, *T. gondii* body). All samples were stained for confocal IFA of host TfR1 and parasite PV-embedded Gra5. (**A**) Shown are representative confocal images for Gra5, TfR1, and merged fluorescence and corresponding distribution plots for Triton X-100 permeabilized cells (top row), saponin permeabilized cells (middle row), and digitonin permeabilized cells (bottom row). (**B**) Comparisons of TfR1 MFI in the total image area and parasite-associated area, grouped by subcellular localization according to permeabilization type. The graph shows the centralized mean and standard deviation denoted in error bars of data pooled from two technical repeats and analyzed by two-way ANOVA (*f* = 17.91, df = 178). (**C**) Comparison of TfR1 ratio (*T. gondii*/total) across subcellular locations according to permeabilization type. Data pooled from two technical repeats and analyzed by ordinary one-way ANOVA (*f* = 111.5, df = 90) are graphically represented by the centralized mean, and standard deviation is denoted by error bars.

**Fig 7:**
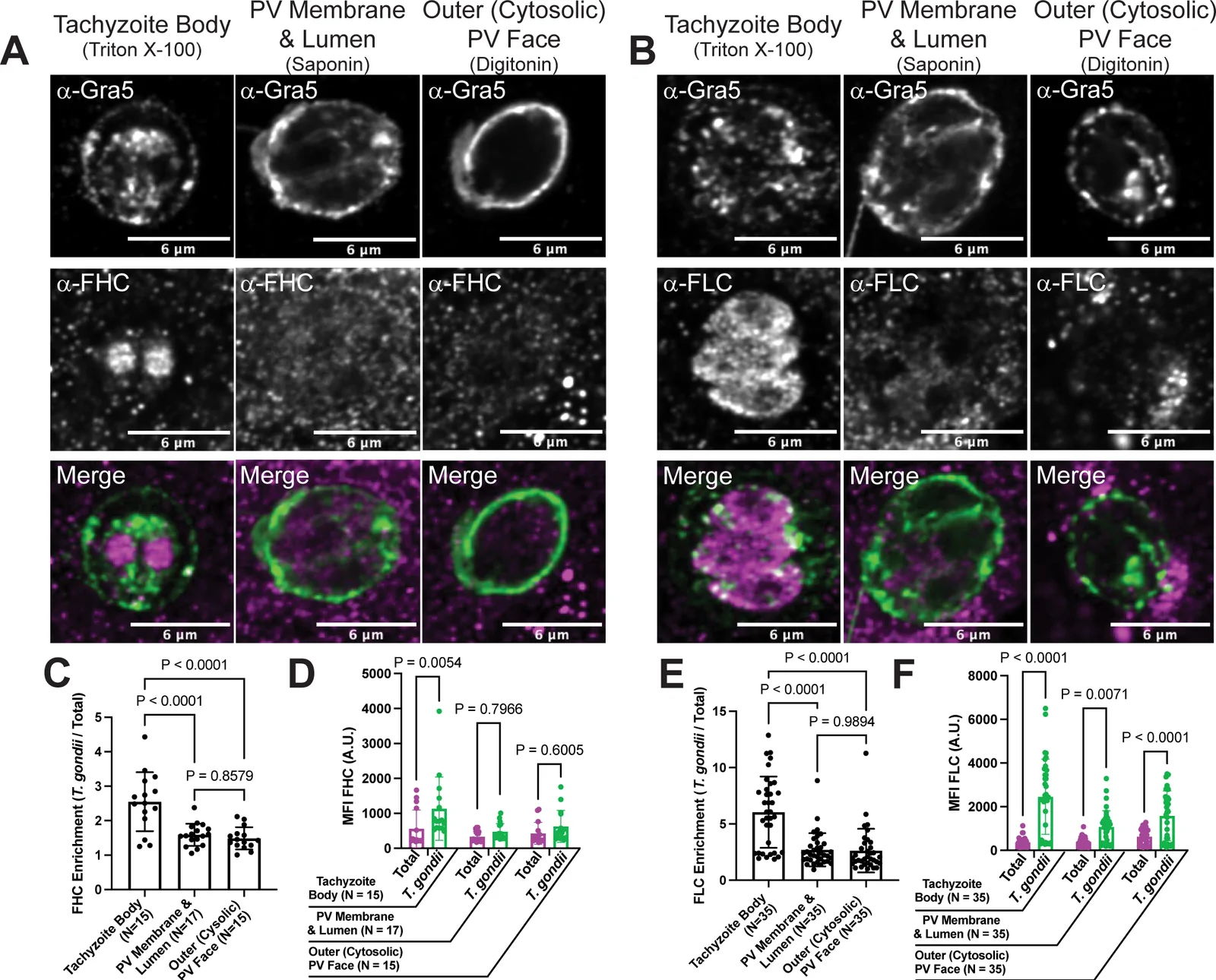
Ferritin heavy and light chains are incorporated within the parasite body. RH strain *T. gondii* 24-hour infections of MRC5 cells were differentially permeabilized to differentially reveal subcellular compartments (digitonin, outer PV leaflet; saponin, PV membrane and lumen; Triton X-100, *T. gondii* body). All samples were stained for confocal IFA of host FHC or FLC and parasite PV-embedded Gra5. (**A**) Representative FHC-IFA images of samples permeabilized with Triton X-100 (left column), saponin (middle column), or digitonin (right column). (**B**) Representative FLC-IFA images of samples permeabilized with Triton X-100 (left column), saponin (middle column), or digitonin (right column). (**C**) Ratio of PV/total FHC MFI between subcellular locations. Analysis performed by ordinary one-way ANOVA (*f* = 17.17, df = 46). (**D**) Comparisons of total and PV-associated FHC grouped by subcellular locations. Analysis performed by two-way ANOVA (*f* = 6.846, df = 40). (**E**) Ratio of PV/total FLC MFI across subcellular locations. Analysis performed by ordinary one-way ANOVA (*f* = 24.55, df = 106). (**F**) Comparisons of total and PV-associated FLC grouped by subcellular locations. Analysis performed by two-way ANOVA (*f* = 9.706, df = 206). All data were pooled from two technical repeats.

To categorize how host proteins redistribute relative to the parasite body, PV lumen, or on the cytosolic face of the PV membrane, we alternatively permeabilized cells for IFA. The differentiation of these subcellular compartments is possible using (i) Triton X-100, for deep IFA staining of the parasite body (54); (ii) saponin, which allows for IFA detection of the PV membrane and lumen but not *T. gondii* cytosol (55); or (iii) digitonin, limiting IFA detection to the surface of the PV (56). Using these agents of variable penetrative depth, we found that it is possible to differentially illuminate deeper structures. Because *T. gondii* dense granule protein Gra5 is embedded in the PV membrane (55), costaining of Gra5 was used as a reference point of the PV boundary. TfR1, FHC, and FLC staining was occluded after saponin or digitonin permeabilization (Fig. 6A, TfR1; Fig. 7A and B, FHC-FLC) compared to Triton X-100. Regardless of the permeabilization method, the MFI of TfR1 and FLC was increased at the parasite location compared to surrounding areas (Fig. 6B and Fig. 7F). To compare the preferential staining in one subcellular compartment over another, we assessed the relative magnitude of TfR1, FHC, and FLC MFI enhancement for each permeabilization method. Using this ratio analysis, we observed that TfR1, FHC, and FLC signals were more greatly enhanced in samples permeabilized by Triton X-100 rather than saponin or digitonin (Fig. 6C and Fig. 7C and E). These results indicate that TfR1, FHC, and FLC transverse the PV to localize and accumulate within tachyzoite bodies.

Identification of host proteins associated with *T. gondii* could pinpoint which iron-related pathways are the root of the parasite iron acquisition. Transferrin receptor 1 is enriched at the host-cytosolic PV interface (57, 58), and exogenously pulsed transferrin is detected within tachyzoite rhoptries (59); thus, the PV barrier may be more selectively breached than previously thought. To further investigate our IFA results, we performed mass spectrometry proteomic analysis of purified RH strain tachyzoites to determine if the host iron homeostasis machinery proteins were present in the body of the parasite. Unsurprisingly, *T. gondii* major secretory proteins (e.g., Gra1, MIC2, and ROP5) were detected, as well as cellular iron transporters VIT and ZRT (S1). However, over 2,000 host proteins were also detected in purified tachyzoites (S1). Among them were peptide fragments with perfect homology to host iron regulatory proteins TfR1, FHC, FLC, ZIP14, HFE, and TfR2 (S2). None of these detected peptides were perfectly conserved to any other human proteins or any strain of *T. gondii* identified or predicted protein. The range of peptide coverage spanned the length of TfR1, FHC, and FLC (Fig. S2 and S2), therefore, TfR1, FHC, and FLC were positively identified within the purified *T. gondii* proteome and were not of parasite origin.

Given the unexpected nature of observing host iron-related machinery in *T. gondii* bodies, we retroactively searched for evidence of this phenomenon in other infections. Previously, *Plasmodium* extract could bind TfR1 antibodies as well as Tf protein, demonstrating evidence of a parasite-encoded TfR capable of binding transferrin (60). However, like *T. gondii*, we were unable to find homology to human iron transport or storage proteins in *Plasmodium* or any other member of the Apicomplexa phylum, besides DMT1 (Table 2).

**TABLE 2:** List of apicomplexan reference genomes assessed for conservation of mammalian iron transport and storage genes

	DMT1	NRAMP1	Ferroportin	Ftn (heavy)	Ftn (light)	Hepcidin	HFE	TfR2	TfRC	Transferrin
Reference organism	*M. musculus*	*M. musculus*	*M. musculus*	*H. sapiens*	*H. sapiens*	*H. sapiens*	*H. sapiens*	*H. sapiens*	*H. sapiens*	*M. musculus*
NCBI accession no.	NP_032758.2	NP_038640.2	AAF80987.1	NP_002023.2	NP_000137.2	AAH20612.1	AAB82083.1	NP_003218.2	AAA61153.1	NP_598738.1
*Toxoplasma gondii*	TGME49_ 267270	TGME49_ 267270	–^*a*^	–	–	–	–	–	–	–
*Plasmodium falciparum*	PF3D7_ 0523800	PF3D7_ 0523800	–	–	–	–	–	–	–	–
*Cyclospora cayetanensis*	XP_ 026190030	XP_ 026190030	–	–	–	–	–	–	–	–
*Neospora caninum*	XP_003880847.1	XP_ 003880847.1	–	–	–	–	–	–	–	–
*Eimeria* spp.	–	–	–	–	–	–	–	–	–	–
*Babesia* spp.	–	–	–	–	–	–	–	–	–	–
*Theileria* spp.	–	–	–	–	–	–	–	–	–	–
*Cryptosporidium* spp.	–	–	–	–	–	–	–	–	–	–
*Sarcocystis* spp.	–	–	–	–	–	–	–	–	–	–
*Hammondia* spp.	–	–	–	–	–	–	–	–	–	–

^*a*^ – indicates that no potential candidates with bit scores of >50 and E-values of <10⁻⁵ were identified.

### Host transferrin receptor is inherited by *T. gondii* daughter cells during division

**Fig 8:**
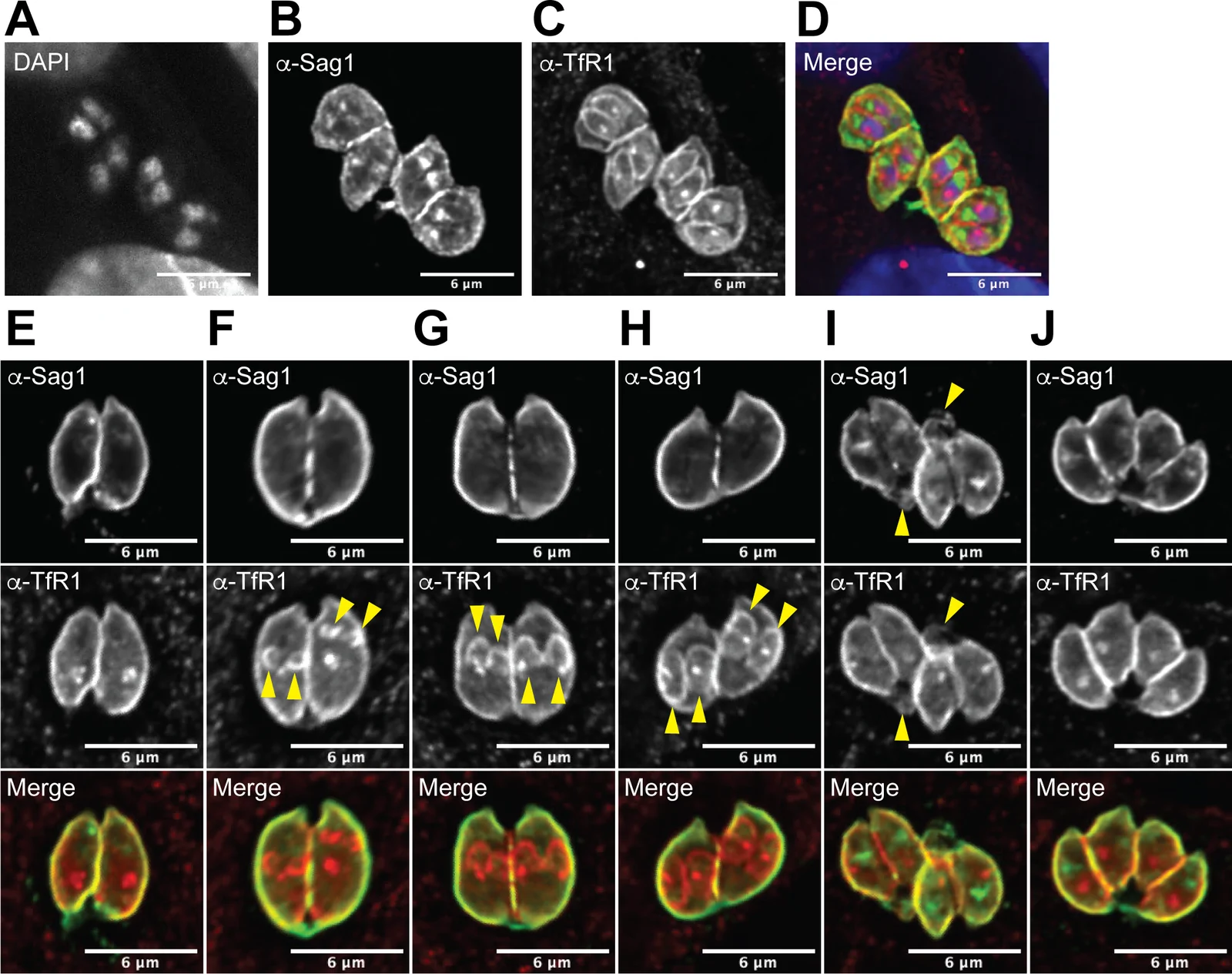
Host transferrin receptor 1 is inherited by daughter cells during endodyogeny. RH strain *T. gondii* infecting MRC5 cells for 15–24 hours was permeabilized with Triton X-100 and stained for confocal Sag1 and TfR1 IFA. The following phenotypes were partially penetrant. (**A–D**) Exemplar images are shown for parasites undergoing endodyogeny by DAPI (**A**), Sag1 (**B**), TfR1 (**C**), and merged (**D**) fluorescence. Additional exemplar images are shown for (**E**) pre-endodyogeny and (**F**) early endodyogeny (arrows indicate morphology associated with initiation of scaffold assembly), (**G**) mid-endodyogeny (arrows indicate morphology associated with scaffold elongation), (**H**) late endodyogeny (arrows indicate morphology associated with organelle segregation), (**I**) cytokinesis (arrows indicate morphology associated with mother cell remnants), and (**J**) post-endodyogeny.

*Toxoplasma* asexual replication is known as endodyogeny (61). We tested the fate of host TfR1 from parent cell to offspring using IFA. Morphology of the parasite as visualized by Sag1 staining showed enlarged tachyzoites undergoing endodyogeny (Fig. 8B). Endodyogeny was confirmed with two segregated nuclei confined within one parental parasite (Fig. 8A). TfR1 immunofluorescence also localized to the periphery of the two offspring cells inside (Fig. 8C). These results phenocopied the distribution of IMC1 during endodyogeny (62). Host TfR1 morphology within newly forming offspring cells closely mirrored each step of endodyogeny (Fig. 8E through J; Movie S3), including the apparent formation of basal buds (Fig. 8F), descending IMC (Fig. 8G), extension to the apical ends (Fig. 8H), and breakdown of the mother cell (Fig. 8I). The deposition of TfR1 early in development suggests that TfR1 acquisition and retainment have a functional purpose for *T. gondii* infection.

### Tachyzoite replication is associated with functional TfR1

**Fig 9:**
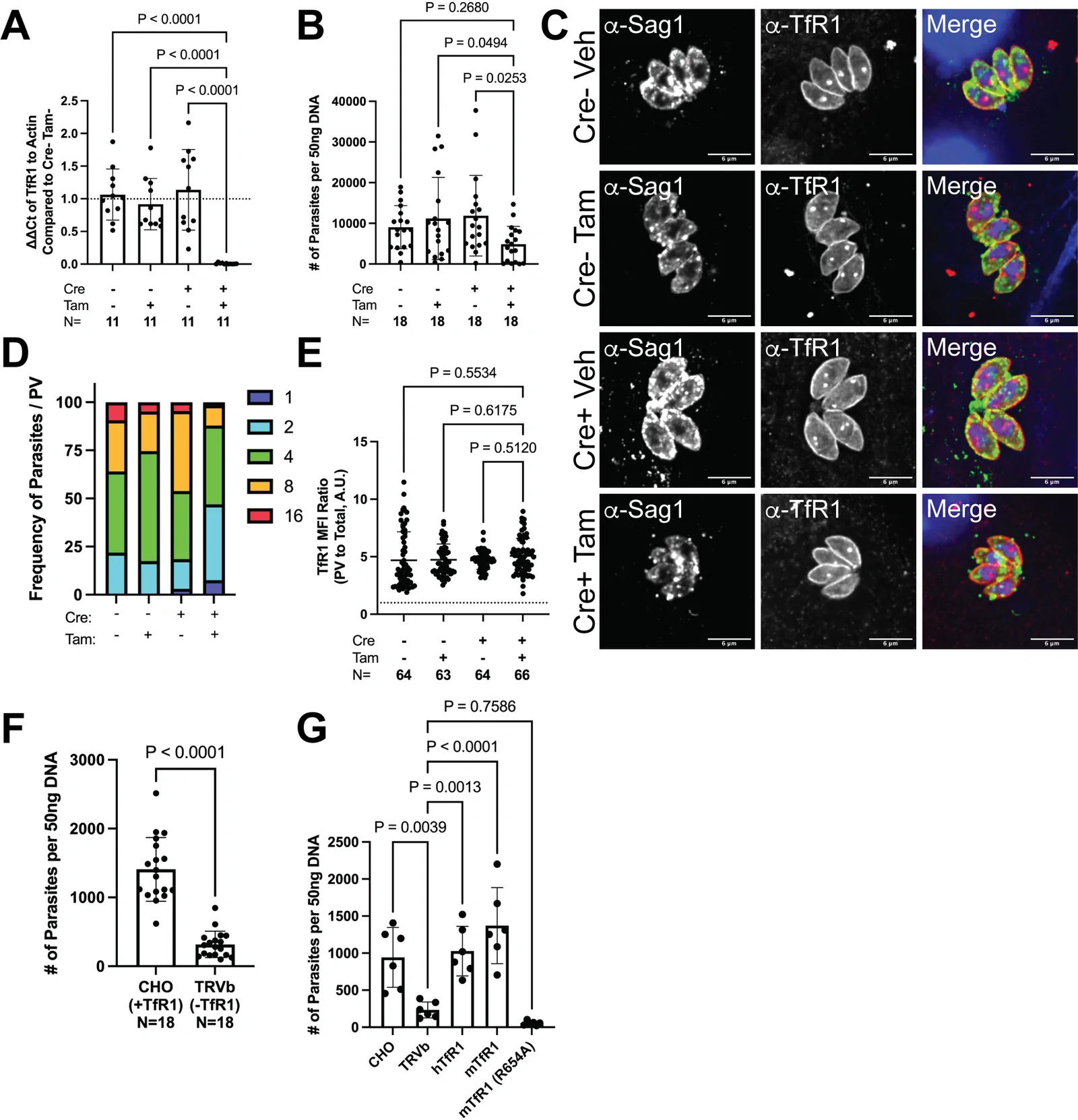
Functional TfR1 is required for successful *T. gondii* infection. TfR1^Flx/Flx^ mice were crossed with the ubiquitin-CreER^T2^ background. Fibroblasts from these mice containing or lacking the Cre transgene were generated. (**A**) Impact of tamoxifen (Tam) or vehicle (Veh) on Cre-possessing or Cre-lacking fibroblast expression of TfR1 mRNA by quantitative reverse transcriptase PCR. The graph depicts the centralized mean and error bars showing standard deviation from data pooled from two technical repeats and analyzed by ordinary one-way ANOVA (*f* = 17.51, df = 43). (**B**) Impact of tamoxifen on *T. gondii* replication in Cre-possessing or Cre-lacking fibroblasts by genome equivalence in real-time PCR. The graph shows data of centralized means and standard deviations in error bars, pooled from three technical repeats and analyzed by ordinary one-way ANOVA (*f* = 2.899, df = 71). (**C**) Representative Sag1, TfR1, and merged IFA images of groups as in panels A and B at 24 hours post-infection. (**D**) Graph shows the distribution of PVs containing 1, 2, 4, 8, or 16 parasites of groups depicted in panel C. Shown proportions were estimated by counting at least 10 isolated images per coverslip from two technical repeats of *n* = 4 each. (**E**) Quantitation of TfR1 MFI of parasite area compared to the TfR1 MFI of the surrounding total area of the groups depicted in panel C. The graphed data depict centralized mean and standard deviation in error bars, pooled from two technical repeats and analyzed by ordinary one-way ANOVA (*f* = 0.8677, df = 256). (**F**) TfR1-deficient TRVb cells and parental CHO cells were infected with RH strain *T. gondii* and assessed for parasite burden. Data were pooled from three technical repeats, analyzed by unpaired Student’s *t*-test (*t* = 9.228, df = 34), and represented by the centralized mean, and error bars show the standard deviations. (**G**) Parental CHO and TfR1-deficient TRVb cells complemented with the human TfR1 gene (hTfR1), murine TfR1 gene (mTfR1), or Tf-binding site mutated murine TfR1 allele (R654A) were infected with RH and assessed for parasite burden as in panel F. The graph depicts the centralized mean, and error bars denote the standard deviation of data representative of three technical repeats and analyzed by ordinary one-way ANOVA (*f* = 16.96, df = 29, *n* = 6).

To explore how host TfR1 expression affects parasite infection, we generated tamoxifen-induced conditional TfR1-deficient (cTfR1KO) murine fibroblasts and infected them *in vitro* with *T. gondii* (Fig. 9). Despite eliminating TfR1 mRNA (Fig. 9A), differences in parasite burden were not significantly different 6 days post-infection comparing cTfR1KO cells to controls (Fig. 9B). Ratios and numbers of 1, 2, 4, 8, and 16 tachyzoite-containing PVs at 24 hours post-infection revealed no differences between Cre− cells ± tamoxifen and Cre+ cells without tamoxifen (Fig. 9C and D), and the difference detected in Cre+ cells with tamoxifen (cTfR1KO) was modest (Fig. 9D). IFA for TfR1 at 24 hours post-infection demonstrated that the parasite still possessed TfR1 with no significant difference in PV TfR1 MFI across all groups (Fig. 9E). These results suggest that nascent TfR1 protein expression by the host cell is not required by the parasite for successful infection.

To further elucidate the functional requirement of TfR1 and Tf for *T. gondii* infection, we obtained TfR1-deficient CHO cells (CHO-TRVb1) generated through artificial selection (63) and CHO-TRVb1 cells complemented with human TfR1 (hTfR), murine TfR1 (mTfR), or murine TfR1 with a R654A point mutation that is unable to bind holo-Tf (R654A) (64), and measured *T. gondii* proliferation *in vitro*. Tachyzoite proliferation was significantly inhibited in the TfR1-deficient CHO-TRVb1 cells (Fig. 9F) compared to wild-type CHO cells. Overexpression of human and murine TfR1 in the CHO-TRVb1 cells restored *T. gondii* proliferation (Fig. 9G). Additionally, the R654A mutated allele was insufficient to rescue parasite growth to wild-type CHO cell levels (Fig. 9G).

**Fig 10:**
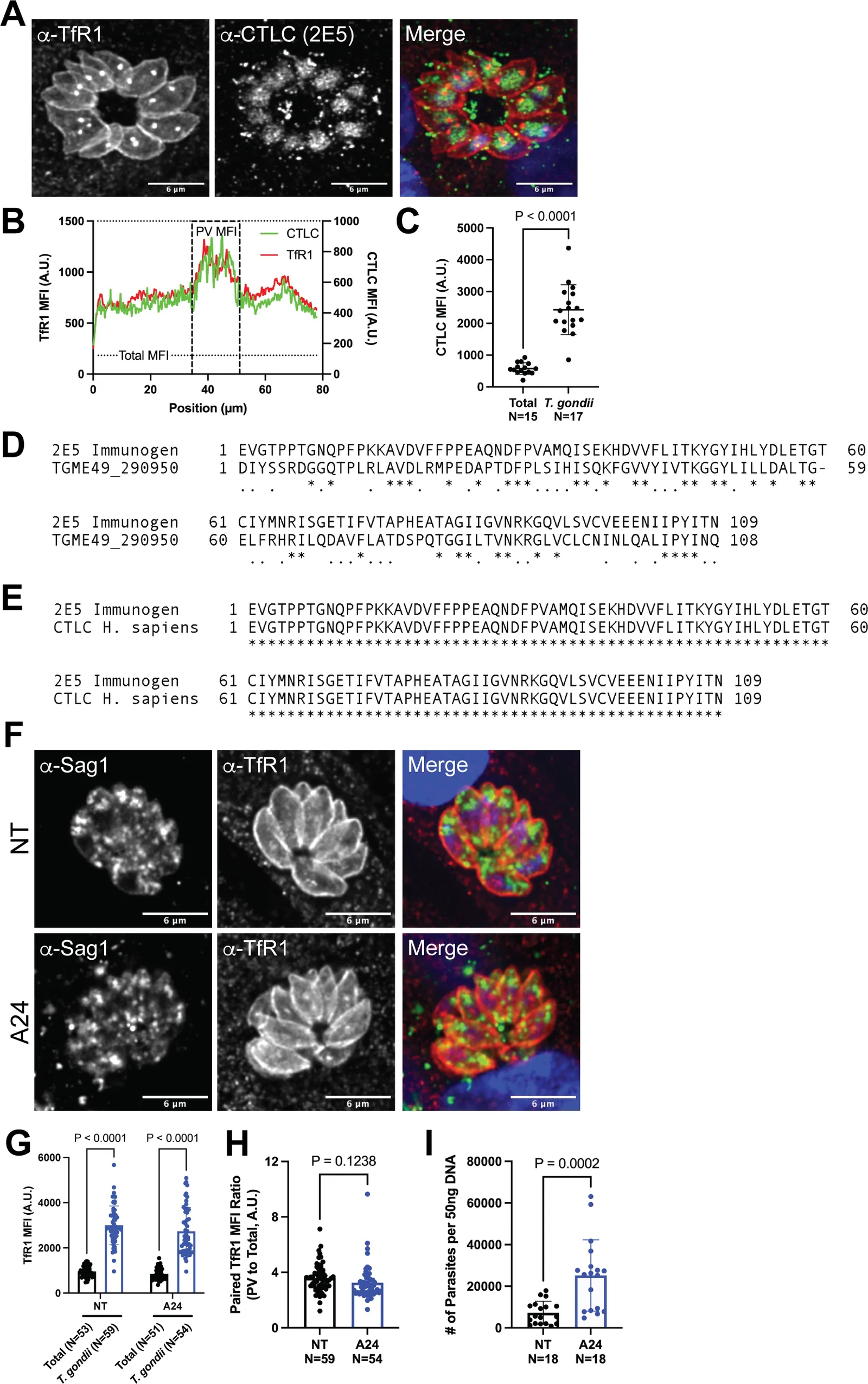
Clathrin light chain associates with *T. gondii*, and blocking endosome recycling confers an advantage to parasite replication. (**A–C**) RH strain *T. gondii* infecting MRC5 cells for 24 hours was permeabilized with Triton X-100 and stained for confocal Sag1 and CTLC IFA. Representative images, distribution plot, and quantitation of parasite association with CTLC are shown. The graph shows centralized means and error bars of the standard deviation of CTLC quantitation pooled from two technical repeats and analyzed with unpaired Student’s *t*-test (*t* = 8.922, df = 30). (**D**) Protein sequence alignment of the epitope used to generate the CTLC clone with the top-scoring homologous *Toxoplasma* gene (TGME49_290950). (**E**) Protein sequence alignment of the epitope used to generate the CTLC clone with the human CTLC gene. (**F–I**) Human MRC5 fibroblasts were treated with TfR1-ERC blocking antibody (A24) and infected with the RH strain *T. gondii*. (**F**) Representative images are shown of 24-hour post-infection IFA for Sag1 and TfR1. (**G**) Comparisons of total- and PV-associated IFA of TfR1 MFI are shown grouped by treatment. The graph depicts the centralized mean and standard deviation in error bars for data pooled from two technical repeats and analyzed by two-way ANOVA (*f* = 3.561, df = 213). (**H**) Comparison of PV/total TfR1 MFI ratio between non-treated and A24-treated groups is shown. The graph depicts the centralized mean and standard deviation in error bars for data pooled from two technical repeats and analyzed by unpaired Student’s *t*-test (*t* = 1.551, df = 111). (**I**) Non-treated and A24-treated MRC5 cells were infected with the RH strain *T. gondii* and assessed for parasite burden by genome equivalence. The graph depicts the centralized mean and standard deviation in error bars for data pooled from three technical repeats and analyzed by unpaired Student’s *t*-test (*t* = 4.229, df = 34).

Growth of *T. gondii* was impaired in TfR1-deficient cells but not in cells when TfR1 transcription was conditionally suppressed, indicating that diversion of existing or recycled TfR1, rather than reprogramming of TfR1 expression, is required for *T. gondii*. Traditionally, TfR1 and Tf uptake occurs by CME and recycling through the endosomal recycling complex (ERC) (31) (65). To define how cellular trafficking pathways of TfR1 and Tf internalization impact *T. gondii*, IFA staining for human clathrin light chain (hCTLC) was performed. *T. gondii* possesses a CTLC ortholog (TGME49_290950); however, tgCTLC diverges extensively at the epitope recognized by the anti-hCTLC clone (2E5, Fig. 10D and E). Anti-hCTLC was observed within the parasite (Fig. 10A through C). To test if TfR1 was being sequestered after CME and before trafficking to the ERC, we utilized antihuman TfR1 clone A24, which enhances CME of TfR1, directs it away from the ERC, and directs it toward degradation in the lysosome (66). Unexpectedly, treatment of human fibroblast cultures with A24 antibody compared to vehicle alone resulted in a nearly fourfold increase (NT: 7,245 ± 5,494, A24: 25,184 ± 17,139) in parasite genomes detected over the non-treated control (Fig. 10I). Despite higher parasite numbers in A24 cultures, TfR1 MFI per parasite was not different from vehicle-alone controls (Fig. 10F through H). These results suggest that misdirecting TfR1 after CME to the lysosome instead of the ERC enhanced parasite proliferation and acquisition of TfR1.

### Theft of TfR1 is dependent on parasite life stage

**Fig 11:**
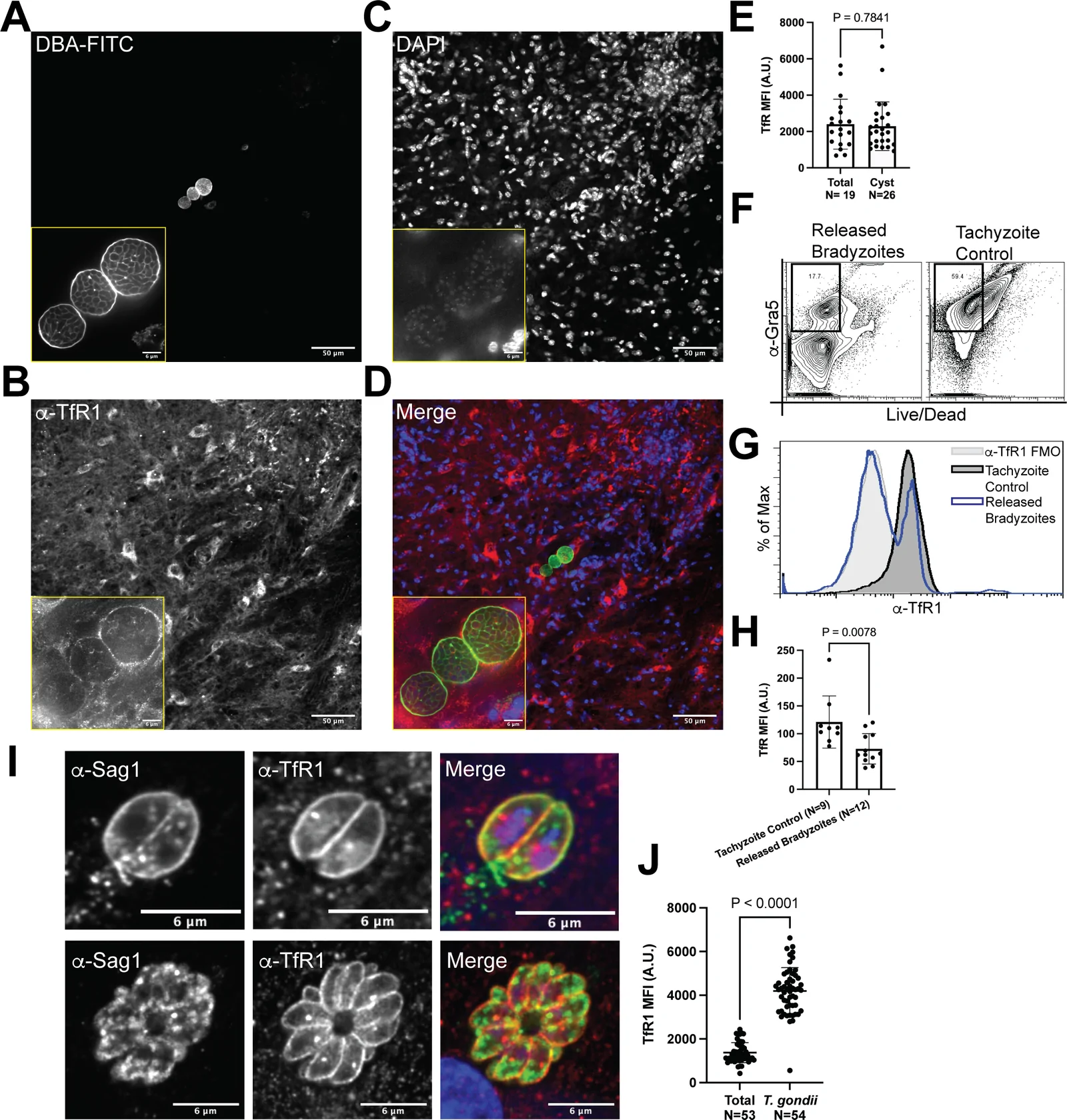
Theft of transferrin receptor 1 is dependent on life stage. (**A–D**) Chronically ME49-strain *T. gondii*-infected C57BL/6 brains were sectioned for IFA staining of *Dolichos bifluorus* agglutinin (DBA) and TfR1. Representative low-magnification and inlaid high-magnification images are shown for DBA (**A**), TfR1 (**B**), DAPI (**C**), and merged (**D**) fluorescence. (**E**) Graph depicting total field or cyst-restricted (boundary of DBA fluorescence) TfR1. MFI represents the centralized mean and standard deviation in error bars. Data pooled from two mice from two independent repeats and analyzed by unpaired Student’s *t*-test (*t* = 0.2758, df = 43). (**F–J**) Cysts purified from chronically ME49 infected mice were trypsinized and assessed for protein expression by flow cytometry or reactivated *in vitro*. (**F**) Displayed are representative dot plots depicting Gra5 expression and viability (Live/Dead negative) of released bradyzoites from cysts or RH tachyzoites grown *in vitro*. (**G**) Representative histogram of TfR1 protein level on live Gra5+ parasites is shown. (**H**) Relative TfR1 MFI on live Gra5+ parasites from the released bradyzoite population or the *in vitro* tachyzoite population is compared. Graphed data pooled from two technical repeats and analyzed by unpaired Student’s *t*-test (*t* = 2.975, df = 19) depict centralized mean and standard deviation in error bars. (**I**) Released ME49 bradyzoites were reactivated in human MRC5 cells for 24 hours, and representative images of Sag1, TfR1, and merged fluorescence are shown. (**J**) Quantitation of the parasite-associated TfR1 of reactivated bradyzoites. Graph represents the centralized mean and standard deviation in error bars for data pooled from two technical repeats and analyzed by unpaired Student’s *t*-test (*t* = 17.71, df = 105).

Immune, nutritional, and environmental stressors trigger tachyzoite conversion into cyst-encased bradyzoites (23, 24, 47, 67). We tested whether bradyzoites within DBA^+^ cysts also harbored host TfR1 in the brain (Fig. 11A). While TfR1 was readily observed on neurons, the accumulation of host TfR1 to parasites was not (Fig. 11B). The MFI of TfR1 in cysts was not significantly different from the surrounding cells or background (Fig. 11E). Live trypsin-released Gra5+ bradyzoites compared to tachyzoite controls were analyzed for host TfR1 by flow cytometry (Fig. 11F and G). Bradyzoites were mostly negative for host TfR1 (Fig. 11G); however, a subpopulation was positive for host TfR1, similar to tachyzoites. Overall, host TfR1 signal was significantly lower in purified (non-reactivated) bradyzoites compared to tachyzoite controls (Fig. 11H). When bradyzoites were reactivated *in vitro*, host TfR1 theft occurred as robustly as strains observed in previous tachyzoite experiments (Fig. 11I and J). These results indicate host TfR1 sequestration occurs more robustly in tachyzoites than in bradyzoites.

## DISCUSSION

*T. gondii* relies on several host-derived nutrients for successful infection, including iron. The interplay of iron homeostasis mechanisms between the host and *T. gondii* is uncertain. Here, we report that apicomplexans lack many homologous genes that could predict a handoff of iron from host to parasite. Instead, during acute infection, *T. gondii* tachyzoites appear to acquire host FHC, FLC, TfR1, and Tf proteins. This process is highly conserved across host cell species and parasite virulence types.

Our results demonstrate *T. gondii* relies on TfR1 binding to Tf for successful infection. Although host CTLC association with tachyzoites suggests that *T. gondii* may intercept clathrin-mediated endocytosis to obtain these proteins, targeting TfR1 for endolysosomal degradation enhanced the parasite’s ability to acquire the receptor and proliferate. Host cells defective in iron uptake through Tf-TfR1 binding yielded low parasite numbers, suggesting that Tf-TfR1 acquisition may carry iron with it. However, iron storage proteins FHC and FLC were observed within the parasite too, highlighting redundant avenues of parasite iron acquisition. Tracking iron from host proteins to the parasite will be informative future research.

Manipulation of host protein is a defining characteristic of viral and parasitic infections, but host protein ingestion, retention, and donation to offspring is an emerging host-pathogen interaction. We observe host TfR1 incorporation into newly forming daughter tachyzoites. Advances in proteomics reveal that virions such as those of influenza virus contain wide arrays of peptides derived from the host (68). Once thought to be artifactual incorporation during the viral lytic cycle, growing evidence suggests that the theft of host protein may be actively functional for pathogen-specific functions such as immune evasion (69).

Heme scavenging for iron by *Plasmodium* can be complemented by transferrin (70). *Plasmodium* extracts contained a transferrin-binding protein indicative of a putative parasite-encoded transferrin receptor (60). *Plasmodium* TfR1 exhibited acylation distinct from human TfR1 (71), and the existence of this protein is still under debate (72). Here, we present no TfR1 homologous sequence in any apicomplexan genome in opposition to the hypothesis that there exists a *Plasmodium* TfR. The Apicomplexa phylum generally lacks other conserved iron transport and storage machinery. Theft of host iron machinery by *T. gondii* thus could represent a conserved strategy across all Apicomplexa.

There could be several mechanisms by which *T. gondii* is stealing host iron machinery. For cytosolic proteins FHC and FLC, *T. gondii* might hijack host ESCRT-II proteins (6, 73). Entry of host cytosolic cargo into tachyzoites may also result from the endocytosis function of the tachyzoite micropore (7, 8). Host cell transmembrane proteins such as TfR1 and Tf are excluded from the PVM during invasion (4, 74, 75). However, soon after, host-derived vesicles are detected within the PV lumen in a Rab11a-dependent manner (76), and exogenously added transferrin particles are detected by transmission EM in the rhoptries of tachyzoites (59). Normally, after clathrin-mediated endocytosis, the Tf-TfR1 vesicle is acidified and either degraded by the endolysosome or recycled in the ERC as dictated by Rab11a and Rab11b (32, 65, 77–80). TfR1 and its associated vesicular trafficking proteins have been preferentially detected at the PVM interface by various methods (57, 58, 75, 76, 81), and thus, host-derived vesicles could represent another mechanism of acquisition. In uncommon events, we observed foci of TfR1 within the PV lumen that bore similarity to Rab11a foci (81), opening the possibility that TfR1 theft may occur through Rab11a modulation. Our results also reveal *T. gondii* ingests host-CTLC, which might indicate that rerouting of host endosomes occurs prior to acidification or recycling. Enhancing TfR1 trafficking to the endolysosome with A24 antibody resulted in increased parasite replication and TfR1 acquisition. Taken together, our results suggest host Tf-TfR1 theft occurs as rerouting of endolysosomes prior to recycling.

Conversion to the bradyzoite life stage in immune-privileged sites is a means of immune evasion that is not afforded to tachyzoites. In addition to differential antigen expression by bradyzoites, the cyst wall is thought to contribute to immune evasion by acting as an additional barrier against the immune response. Tachyzoites likely possess other means of immune evasion, as conversion-deficient strains are still not cleared and cause mortality (47, 82). Interestingly, bradyzoites, which replicate rarely or slowly, did not significantly accumulate host TfR1. This may be a reflection of the nutritional sensing of iron contributing to life-stage conversion, as iron starvation induces life-stage conversion (23, 24) and BFD-1 shares homology to the iron-sensing Myb2 protein of *Arabidopsis thaliana* (Table S1). However, the accumulation and persistence of TfR1 in tachyzoites, but not bradyzoites, might also correspond to the different mechanisms of the two life stages regarding immune evasion. Recently, it was shown that *T. gondii* tachyzoites bind host PM protein CD36, and the host CD36 protein is required for parasite attachment and invasion of macrophages. Moreover, in the absence of host CD36, there was increased mortality of mice due to extensive tissue damage (83). Therefore, it is plausible that theft of FHC, FLC, TfR1, and Tf, or tachyzoite binding of other host proteins such as CD36, may provide a cloak of host antigen associated with the tachyzoite PM as a mechanism to circumvent non-self-recognition to prevent killing of host cells. Potential therapies that could block host cloaking would be effective in preventing parasite escape.

## Data Availability

The mass spectrometry proteomics data have been deposited to the ProteomeXchange Consortium via the PRIDE (84) partner repository with the data set identifier PXD084269.

## SUPPLEMENTAL MATERIAL

Data S1 mbio.01248-26-s0001.xlsx MS results.

Data S2 mbio.01248-26-s0002.xlsb Peptide ID from MS.

Supplemental material mbio.01248-26-s0003.pdf Supplemental text, figures, tables, and movie legends.

Movie S1 mbio.01248-26-s0004.avi Theft of transferrin receptor 1 by *T. gondii.*

Movie S2 mbio.01248-26-s0005.avi Theft of transferrin by *T. gondii.*

Movie S3 mbio.01248-26-s0006.avi Inheritance of host TfR1 by *T. gondii* and daughter cells during endodyogeny.

ASM does not own the copyrights to Supplemental Material that may be linked to, or accessed through, an article. The authors have granted ASM a non-exclusive, world-wide license to publish the Supplemental Material files. Please contact the corresponding author directly for reuse.
