# Supplementary material for: Theft of host transferrin receptor-1 by Toxoplasma gondii is required for infection
Source: mBio. 2026 Oct 23;17(11):e01248-26. doi: 10.1128/mbio.01248-26 (PMC13753791; doi:10.1128/mbio.01248-26)
Supplement: Supplementary file 1 [file mbio.01248-26-s0003.pdf]

Supplementary Information for:

Theft of Host Transferrin Receptor-1 by *Toxoplasma gondii* is Required for Infection

Stephen L. Denton[1], Alexa Mejia[1], Lindsay L. Nevarez[1], Miguel P. Soares[2], Barbara A. Fox[3], David J. Bzik[3], and Jason P. Gigley[1]*.

[1]Department of Molecular Biology, University of Wyoming, Laramie, WY, USA

[2]Instituto Gulbenkian de Ciência, Lisbon, Portugal

[3]Department of Microbiology and Immunology, Geisel School of Medicine at Dartmouth, Lebanon, New Hampshire, USA

*Address correspondence to Jason Gigley, jgigley@uwyo.edu

RESOURCE AVAILABILITY

Lead contact

Further information and requests for resources and reagents should be directed to and will be fulfilled by the lead contact, Jason P. Gigley (jgigley@uwyo.edu).

Materials availability

The Ubiquitin-CreER^{T2} and littermate control mouse lines will be available upon request to the lead contact. No other unique reagents or models were generated by this study.

Data and code availability

No unique code was generated by this study. All raw data used for analysis in this paper will be provided upon request to the lead contact.

EXPERIMENTAL MODEL AND STUDY PARTICIPANT DETAILS

Tissue culture

MRC5 Human Lung Fibroblasts (RRID:CVCL_0440) were maintained in Complete Media (Dulbecco's Modified Eagles Medium with 4.5g/L glucose, L-glutamine, and sodium pyruvate, supplemented with 1X Penicillin/Streptomycin, Amphotericin B, and 10% Fetal Bovine Serum) at 37˚C and 5% CO_2. Cells were expanded when 85% confluent by detaching the cells with 0.05% Trypsin- EDTA and expanding cells at a ratio of 1:4 in terms of surface area available to the monolayer.

Chinese Hamster Ovary (CHO, RRID:CVCL_0214) and CHO-derived cell lines were maintained in CHO Media (Ham's F-12 Media supplemented with 1X Penicillin/Streptomycin, 2mg/mL D-Glucose, and 3% FBS) at 37˚C and 5% CO_2. CHO-derived TRVb cells were kindly gifted by Caroline A. Enns, Oregon Health & Science University. TRVb-derived hTfR, mTfR, and mTfR-R654A complemented cells, kindly gifted by Paul J. Schmidt, Boston Children's Hospital and Harvard School of Medicine, were supplemented further with 1X Geneticin. Cells were expanded when 85% confluent by detaching the cells with 0.05% Trypsin-EDTA and expanding the cells at a ratio of 1:3.

Murine Fibroblasts (MFs) were isolated from male and female C57BL/6J mice (RRID:IMSR_JAX:000664) adapted from established protocols [34]. Briefly, 1cm-diameter sections from both ears of humanely euthanized mice were sterilized by submersion in 70% ethanol for 5 minutes and air dried in a sterile petri dish. The sections were digested at 37˚C for 90 minutes in 2mL Digestion Buffer (2.5mg Pronase and 5mg Collagenase D in Isocove's Complete Media). Cells were released from tissue by straining through a 70µm cell strainer and washed in 10mL MF Media (Isocove's DMEM with L-Glutamine and 25mM HEPES supplemented with 1X Penicillin/ Streptomycin, Amphotericin B, Non-Essential Amino Acids, 0.1mM Sodium Pyruvate, 0.2mM Glutamine XL, 10% Fetal Bovine Serum, and 0.1mM 2-mercaptoethanol) before seeding into tissue-culture treated plates. Cells were regularly maintained in MF Media at 37˚C and 5% CO_2 and expanded when 80% confluent by detaching the cells with 0.05% Trypsin with EDTA, expanding cells at a ratio of 1:4 in terms of surface area available to the monolayer. MFs isolated from Ubiquitin-CreER^{T2} experiments were isolated from 3 month old male mice.

Type I Strain RH (ATCC: 50174) and Type II Strain Prugniaud (a kind gift from Dr. David J. Bzik, Dartmouth) *Toxoplasma gondii* parasites were regularly maintained by regular passage upon lysis in MRC5 fibroblasts at an MOI of ~0.5 in Black Media (Dulbecco's Modified Eagles Medium with 4.5g/L glucose, L-glutamine, and sodium pyruvate, supplemented with 1X

Penicillin/Streptomycin, Amphotericin B, and 1% Fetal Bovine Serum) at 37˚C and 5% CO_2. 10,000 Prugniaud strain tachyzoites were infected intraperitoneally into C57BL/6J mice every 10-12 *in vitro* passages, and re-isolated from the mouse brains to ensure maintenance of the cyst-forming ability of this strain of parasite. Type II Strain ME49 (ATCC: 50611) was routinely passaged by cyst isolation from 5 week-post-infected CBA/J mice brains and subsequent inoculation of 10 cysts intragastrointestinally.

All tissue culture experiments were performed when fibroblasts reached 100% confluency. Where appropriate, (Z)-4-hydroxy Tamoxifen (Cayman Chemical Company #14854) was dissolved in DMSO at 2.5mM and added to cultures to a final concentration of 2.5μM at the indicated timepoints. For A24 monoclonal antibody blockade experiments, A24 (Creative Biolabs #TAB-622CT) was administered to cultures at 1μg/mL every two days starting 15 minutes before infection.

Animal Maintenance

All animal husbandry was performed by the laboratory of Jason P. Gigley at the University of Wyoming and in accordance with the guidelines approved by the University of Wyoming Institutional Animal Care and Use Committee. Mice were housed at room temperature at maximum 5 mice to a cage in a HEPA-filtered and air flow-controlled Animal Care Systems OptiMice® tower. Mice were fed autoclavable 18% Protein Teklad Rodent Chow (Teklad 2018SC) and water *ad libitum.* With the exception of genotyping purposes, mice were experimentally naïve prior to any experimentation. C57BL/6J (RRID:IMSR_JAX:000664) and Ubiquitin-CreER^{T2} (RRID:IMSR_JAX:007001) mice were purchased from Jackson Laboratories. TfR1^{Flx} mice were a kind gift from Mark Andrews, Harvard University. Mouse genotypes were assessed at 4-6 weeks of age from genomic DNA isolated from ear clips *via* digestion in DirectPCR Digestion Buffer + Proteinase K (Viagen Biotech) using the following primer pairs: Ubiquitin-CreER^{T2} Forward (0.5μM PCR Reaction): 5'- GAC GTC ACC CGT TCT GTT G -3', Ubiquitin-CreER^{T2} Reverse (0.5μM PCR Reaction): 5'- AGG CAA ATT TTG GTG TAC GG -3', and TfR^{Flx} Forward (0.3μM PCR Reaction): 5'-TTC AGT TCC CAG TGA CCA CA -3', TfR^{Flx} Reverse (0.3μM PCR Reaction): 5'- TCC TTT CTG TGC CCA GTT CT -3', where the presence of the Ub-CreER^{T2} transgene was indicated by a 475nt agarose gel band, the wild-type TfR1 gene was indicated by a 165nt agarose gel band, and the floxed TfR1 gene was indicated by a 310nt agarose gel band. Female mice were used at 8-12 weeks of age for cyst isolation and reactivation studies; the influence of sex may be a limitation of these studies.

METHOD DETAILS

Cyst Isolation and Reactivation

Cyst isolation was adapted from a protocol described previously [36]. Chronically infected mice were humanely euthanized by CO_2 inhalation and dissected with sterile instruments to remove the brain tissue, which was subsequently homogenized with a Dounce tissue grinder in PBS. Cysts were manually enumerated by sampling 10μL aliquots of brain homogenate under a light microscope. Cysts were further purified on a 90/40/20% Percoll gradient in 1X PBS and retrieved from the 90/40 interface following differential centrifugation at 900 RCF for 20 minutes at 21˚C with no brake. Bradyzoites were released by suspension in 100μL 0.05% Trypsin-EDTA for 30 seconds, quenched in Black Media, and immediately inoculated into *in vitro* cultures or subjected to flow cytometry protocol. For microscopic evaluation, reactivated parasites were subjected to confocal microscopy methodology 24-36 hours after *in vitro* inoculation.

Brain Sectioning

Brain sectioning protocol was adapted from methods described previously [37]. C57BL/6 mice chronically infected with ME49 strain *T. gondii* were anesthetized with a barbiturate cocktail and exsanguinated *via* perfusion. Perfusion was performed by a peristaltic pump; the 27G needle outlet was inserted into the left ventricle of the heart, an incision was made through the right atrium, and the pump operated at a rate of 10mL / minute. Perfusion occurred first with 20mL of saline (25U Heparin / mL, 0.9% (w/v NaCl), then with 100mL of fixative (4% w/v Paraformaldehyde in 0.1M phosphate buffer). The mouse corpse was incubated at 4˚C for 24h in fixative, and the whole brain incubated a further five days in cryopreservative (10% v/v glycerol, 2.5% v/v dimethyl sulfoxide in 0.1M phosphate buffer). The brains were mounted in OCT solution and affixed to a microtome by freezing with dry ice. 40μm sections of brain were transversely collected and stored at 4˚C in 0.1M phosphate buffer until prepared for microscopic evaluation.

Confocal Microscopy (CM)

Coverslips (#1 Thickness German Glass, 15mm Diameter) were individually washed in a 1N HCl solution for 24 hours. The coverslips were individually and subsequently rinsed in 70%, 80%, and 90% ethanol solutions before storage in 95% ethanol. Fibroblasts were inoculated onto prepared and dried coverslips upon expansion into a 24-well format. Immediately upon reaching confluency on the coverslips, fibroblasts were inoculated with 1,000 freshly lysed parasites per coverslip for 24 hours.

Infected coverslips were fixed, permeabilized and blocked, and stained for immunofluorescence (IFA) in the culture wells before mounting on a microscope slide. Fixation occurred in 4% Paraformaldehyde (w/v) in PHEM buffer (60mM PIPES, 27mM HEPES, 10mM EGTA, and 8.25mM $MgSO_4$) for 10 minutes at ambient temperature. The fixative was washed away and residual fixative was quenched in 0.1M glycine. The cells were permeabilized and blocked in PHEMBlock (3% FBS and 0.2% Triton X-100 in PHEM buffer) for 30 minutes at room temperature. Host proteins were first stained by incubation with indicated rabbit IgG in PHEMBlock for 16 hours at 4˚C in the dark, then counterstained with goat IgG secondary antibody in PHEMBlock for 8 hours at 4˚C in the dark. Parasite proteins or alternative host proteins were stained next by incubation with indicated IgG in PHEMBlock for 16 hours at 4˚C, followed by counterstain with indicted IgG secondary antibody or streptavidin-conjugated fluorophores in PHEMBlock for 8 hours at 4˚C in the dark. After staining was complete, coverslips were washed once in 2μM DAPI in PBS and twice in PBS. Coverslips were mounted on microscope slides with Prolong Gold Antifade Mountant with DAPI (ThermoFisher P36931).

For alternative permeabilization experiments, coverslips were prepared, seeded, inoculated, fixed, and quenched as above. Permeabilization and blocking occurred by a 30 minute incubation in 0.2% (v/v) Triton X-100, 0.01% (w/v) Saponin, or 0.005% (w/v) in 3% FBS PHEM. All staining steps occurred identically to above except the antibodies were diluted in 3% FBS PHEM without permeabilization agent.

Fixed brain sections were blocked in blocking buffer (3% FBS and 0.1% Triton X-100 in PBS) for one hour and stained with concurrent DBA-FITC and α-TfR1 (JF0956) in blocking buffer overnight at 4˚C. Sections were washed three times with PBS and secondarily stained with goat a-rabbit secondary antibody in blocking buffer for eight hours at 4˚C. Brains were washed with 2μg/mL DAPI in PBS once, and twice in PBS before mounting on a microscope slide with Prolong Glass Antifade Mountant (ThermoFisher P36984).

All samples were imaged with an Olympus IX81 confocal microscope on Metamorph v7.8.6.0 software and analyzed with ImageJ software v1.53f51. Images may have modified brightness and contrast for presentation purposes. For quantification purposes, at least 5 isolated fields were quantified per experimental condition per technical repeat.

Parasite Burdens by Quantitative PCR (qPCR)

Fibroblast lines were inoculated with *T. gondii* according to host cell type; MRC5, MF, or CHO lines were infected with 100 tachyzoites for six days in Black Media. Host cells and parasites were lifted with a cell scraper and pelleted by centrifugation. Genomic DNA was purified from the samples using the DNeasy Blood & Tissue Kit (Qiagen 69506) and normalized to 6.25ng/µL. A standard curve of known quantity of RH gDNA was assessed alongside 50ng of sample DNA as template to query for the *T. gondii* unique gene B1 using SSO Advanced SYBR Master Mix (Bio-Rad 1725017) and 0.5µM primers (F: 5'- CGT CCG TCG TAA TAT CAG -3', R: 5'- GAC TTC ATG GGA CGA TAT G -3') [38]. The thermocycler conditions were 1: 95˚C for 10 minutes, 2: 95˚C for 5 seconds, 3: 60˚C for 5 seconds, 4: 72˚C for 5 seconds + plate read, 5: Cycle 2-4, 44 repetitions, 6: Melt Curve 65˚C to 95˚C with 0.5˚C, 5 second increments + plate read.

Quantitative Reverse Transcriptase PCR (qRT-PCR)

Samples were dissolved in TRI reagent (Sigma-Aldrich T9424), further disrupted in a tissue grinder, and phase-separated by addition of chloroform in a 1:5 ratio. The aqueous RNA was precipitated in isopropanol and washed in 75% ethanol, air-dried, and resuspended in DEPC-Treated water supplemented with 1X RNaseOUT (Invitrogen 10777019). RNA samples were further purified by the RNA Cleanup protocol of RNeasy Kit (Qiagen 74104). 1µg of RNA samples were used as template in cDNA generation using Oligo d(t) primers and Superscript IV (Invitrogen 18091050) according to manufacturer instructions. PCR of the resulting cDNA was performed with SSO Advanced SYBR Master Mix (Bio-Rad 1725017) and 0.5µM of primers against mouse actin (F: 5'- ACC CAC ACT GTG CCC TAC TA -3', R: 5'- CAC GCT CGG TCA GGA TCT TC -3') [39] and mouse TfR1 (F: 5'- TCA AGC CAG ATC AAT TCT C -3', R: 5'- AGC CAG TTT CAT CTC CAC ATG -3') [40]. The thermocycler conditions were 1: 95˚C for 3 minutes, 2: 95˚C for 15 seconds, 3: 60˚C for 15 seconds, 4: 72˚C for 1 minute 20 seconds + plate read, 5: Cycle 2-4, 44 repetitions, 6: Melt Curve 65˚C to 95˚C with 0.5˚C, 5 second increments + plate read. Data were analyzed by the ΔΔCt method.

Western Blot (WB)

Fibroblast lines or lines infected with *T. gondii* at an MOI of 0.1 were incubated in Black Media for two days at 37˚C and 5% CO_2. Cells were detached from the flask with a sterile cell scraper. Only for infected flasks, parasites were isolated by disrupting host cells by twice-passage of samples through a 27G needle and twice-filtration through a 3µm membrane. Every sample was washed twice by centrifugation in PBS, and finally resuspended in Lysis Buffer (20mM HEPES, 10mM Sodium Citrate, 10mM Sodium Pyrophosphate, 50mM Sodium Fluoride, 1mM Sodium Vanadate, 50mM B-Glycerophosphate, 5mM EDTA, and 0.5% NP-40 + fresh addition of 2mM Benzamidine, 0.2mM PMSF, 40µg/mL Leupeptin, 40µg/mL Aprotinin, and 4µg/mL Pepstatin A). Cell lysate was agitated with a tissue grinder and insoluble material was removed by centrifugation. 30µg of purified protein was denatured in SDS Loading Buffer (4% w/v SDS, 20% v/v Glycerol, 0.1M Tris-HCl pH 6.8, 2mg/mL bromophenol blue, and 0.2M DTT) for 10 minutes at 95˚C before loading in a 10% SDS-PAGE Gel (Bio-Rad 4561034). Sample proteins were

separated in 1X SDS Running Buffer (25mM Tris, 20mM Glycine, 0.1% w/v SDS) at 13mA for ~1.5hr until the reference ladder (Bio-Rad 161-0374) was separated. Transfer was completed to a methanol-activated PVDF membrane in Transfer Buffer (20% v/v methanol in 25mM Tris, 192mM glycine) at 300mA and 4˚C for 2hr. Membranes were blocked in 5% (w/v) Nonfat Milk in Wash Buffer (0.1% Tween-20 in PBS) for 30 minutes, washed three times, blotted with primary antibody in Blocking Buffer for 1.5hr at 22˚C, washed three times, blotted with secondary antibody in Blocking Buffer for 1.5hr at 22˚C, and washed 4 times. Immediately before chemiluminescent exposure in an Invitrogen iBright machine, membranes were developed in a solution of equal volumes of ECL peroxidase and luminol reagents (Bio-Rad 170-5060).

Mass Spectrometry

Orbitrap Astral DIA – 40 minutes – CME. RH tachyzoites were grown in vitro in MRC5 fibroblasts as described above and once cultures lysed the host cell monolayer, the parasites were purified by filtration through 3 um pore filtration (Millipore). Tachyzoites were washed 3 times with sterile 1 X PBS and pelleted by centrifugation for 7 minutes at 2200 rpm and 4C. Pellets were then frozen and sent to University of Arkansas for Medical Sciences (UAMS) IDeA National Resource for Quantitative Proteomics facility for analysis. Total protein from each sample was reduced, alkylated, and purified by chloroform/methanol extraction prior to digestion with sequencing grade modified porcine trypsin (Promega). Tryptic peptides were then separated by a reverse phase Ion-Opticks-TS analytical column (25 cm x 75 um with 1.7 um C18 resin) supported by an EASY-Spray nano-source and stabilized with a Heater THOR Controller (Ion-Opticks) at 60°C. Peptides were trapped and eluted from a (PepMap Neo, 300um x 5mm Trap) using a Vanquish Neo UHPLC nano system (Thermo Scientific) which kept the samples at 11°C before injection. Peptides were eluted at a flow rate of 0.350uL/min using a 35 min gradient from 98% Buffer A (Buffer A = 0.1% formic acid, 0.5% acetonitrile in water) :2% Buffer B (Buffer B = 80% acetonitrile, 20% water, 0.1% formic acid) to 94.5:5.5 at 0.1 minutes to 56:44 at 27.1 minutes followed by a column wash of 45:55 at 29.7 minutes to 1:99 at 35 minutes followed by equilibration back to 98:2. Eluted peptides were ionized by electrospray (2.5 kV) followed by mass spectrometric analysis on an Orbitrap Astral mass spectrometer (Thermo). Precursor spectra were acquired from 380-980 Th, 240,000 resolution, normalized AGC target 200%, maximum injection time 3 ms. DIA acquisition on the Orbitrap Astral was configured to acquire 199, 3 Th window from 380-980 Th, 25% HCD Collision Energy, normalized AGC target 100%, maximum injection time 3 ms. Fragment (MS2) scan range from 150-2000 Th with an RF Lens (%) set to 40. Data Analysis: Spectronaut. Following data acquisition, data were searched using Spectronaut (Biognosys version 20.1) against the UniProt Homo sapiens database (Proteome ID: UP000005640, Taxon ID: 9606, 2nd version of 2025) and Toxoplasma gondii database (Proteome ID: UP000001529, Taxon ID: 508771, 2nd version of 2025) using the directDIA method with an identification precursor and protein q-value cutoff of 1%, generate decoys set to true, the protein inference workflow set to maxLFQ, inference algorithm set to IDPicker, quantity level set to MS2, cross-run normalization set to false, and the protein grouping quantification set to median peptide and precursor quantity. Fixed Modifications were set to Carbamidomethyl (C) and variable modifications were set to Acetyl (Protein N-term), Oxidation (M).

Flow Cytometry

Tachyzoites were inoculated into indicated fibroblast culture and later retrieved by mechanical disruption and filtration, similar as in Western Blot protocol. Alternatively, bradyzoites isolated

from cyst isolation and reactivation protocol were retrieved after the trypsinization quench. Parasites were enumerated on a hemacytometer and plated at 10^6 per well in a FACS plate. Viability was assessed by Live/Dead Aqua (Invitrogen L34966), according to manufacturer instructions, prior to fixation in 4% paraformaldehyde in PHEM. There were four separate rounds of antibody staining containing, in order, a-TfR1 JF0956, Goat a-Rabbit IgG Secondary, a-Gra5, and Donkey a-Mouse IgG Secondary in PHEMBlock. Cells were finally resuspended in PBS and processed in Guava EasyCyte 12HT Flow Cytometer Serial #8470120112 and analyzed in FlowJo v.9.7.7 software.

In silico identification of potential T. gondii iron-related genes

Iron-related proteins were manually selected and cataloged for query by literature review. FASTA protein sequences of reference genes identified from primary reports were downloaded from the NCBI Protein Database and queried for homology in the *Toxoplasma gondii* ME49 genome (ToxoDB Toxoplasma Bioinformatics Resource, ToxoDB.org) or the *Plasmodium falciparum* 3D7 genome (PlasmoDB Plasmodium Bioinformatics Resource, PlasmoDB.org) by pBLAST. Potential candidates were limited to those that had bit scores greater than 50 and E-values less than $1.00*10^{-5}$. Identified *T. gondii* genes were then cross-referenced to CRISPR Fitness Scoring [41]. Any protein sequence alignments were performed by ClustalW analysis with a gap penalty of 10.0 in MacVector v17.0.10 software.

Quantification and Statistical Analysis

Data were collected and organized Microsoft Excel v16.54 software. Statistics and graph generation were completed using Graphpad Prism v.9 and Microsoft Excel v16.54 software. Statistics were performed between two groups by Student's t-test, between multiple groups by Ordinary One-Way ANOVA, or between two groups within multiple conditions by Two-Way ANOVA. Relevant statistical values such as sample size (n), P values (p), t-values (t), F-values (F), and degrees of freedom (df) are provided in figures and figure legends. Figures were assembled in Adobe Illustrator 2021, references in Endnote X8.

References

Expanded protocol, Table S1, and Table S2 references are numbered according to their listing in the main article. Table S3 references are numbered according to the reference list included in this supplementary packet.

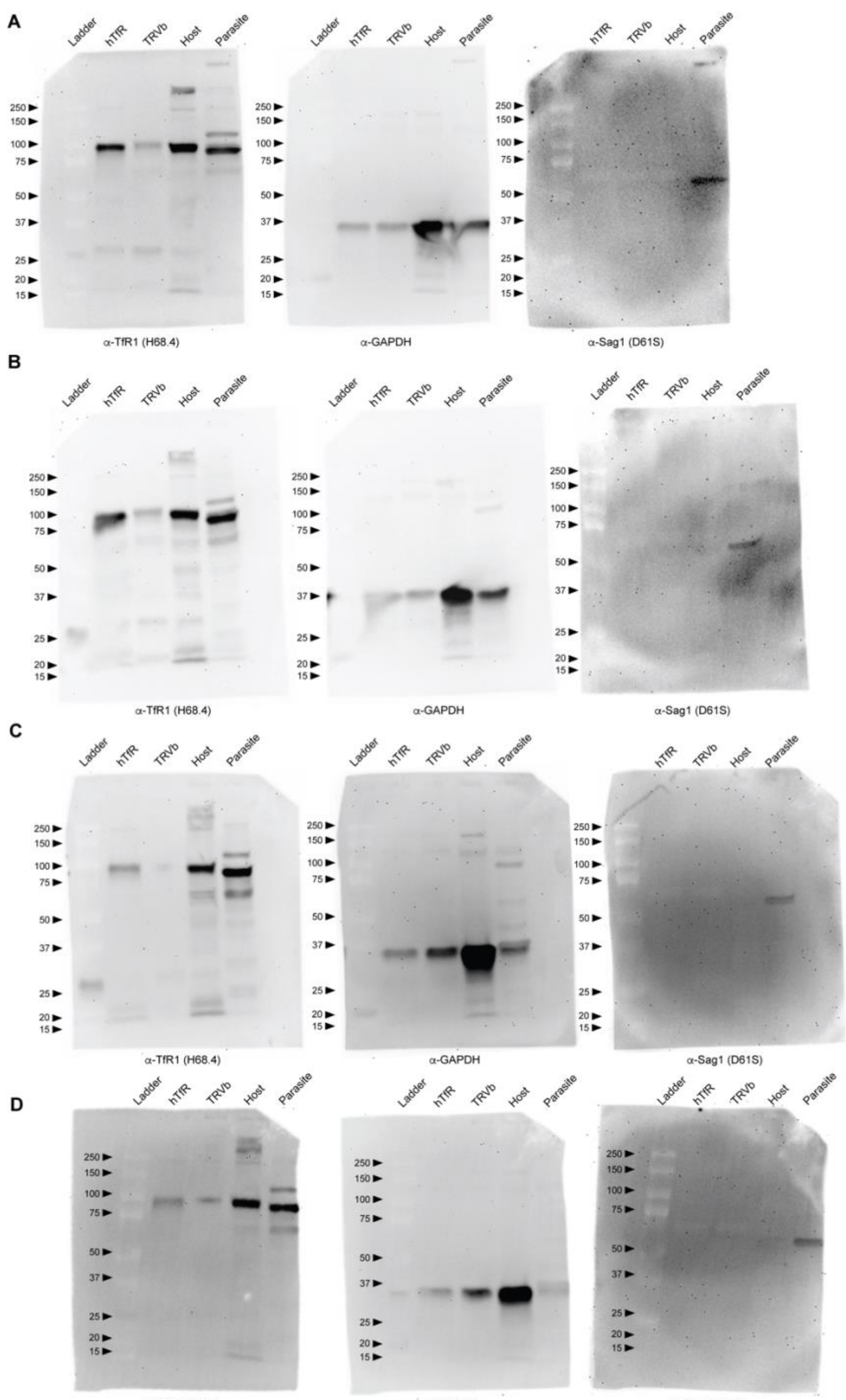

Figure S1. Uncropped western blot images.
30μg of cell lysates were probed in Western Blot for TfR1, Sag1, and GAPDH. Positive control hTfR cell lysates are TfR1-deficient cells reconstituted with the human TfR1 gene. Negative control TRVb cells are the TfR1-deficient cell. The uninfected control is host cell lysate from uninfected MRC5 cells. Purified parasites are RH strain *T. gondii* mechanically lysed from host cells and filter purified. **A-D)** Independent technical repeats for Experiments 1-4.

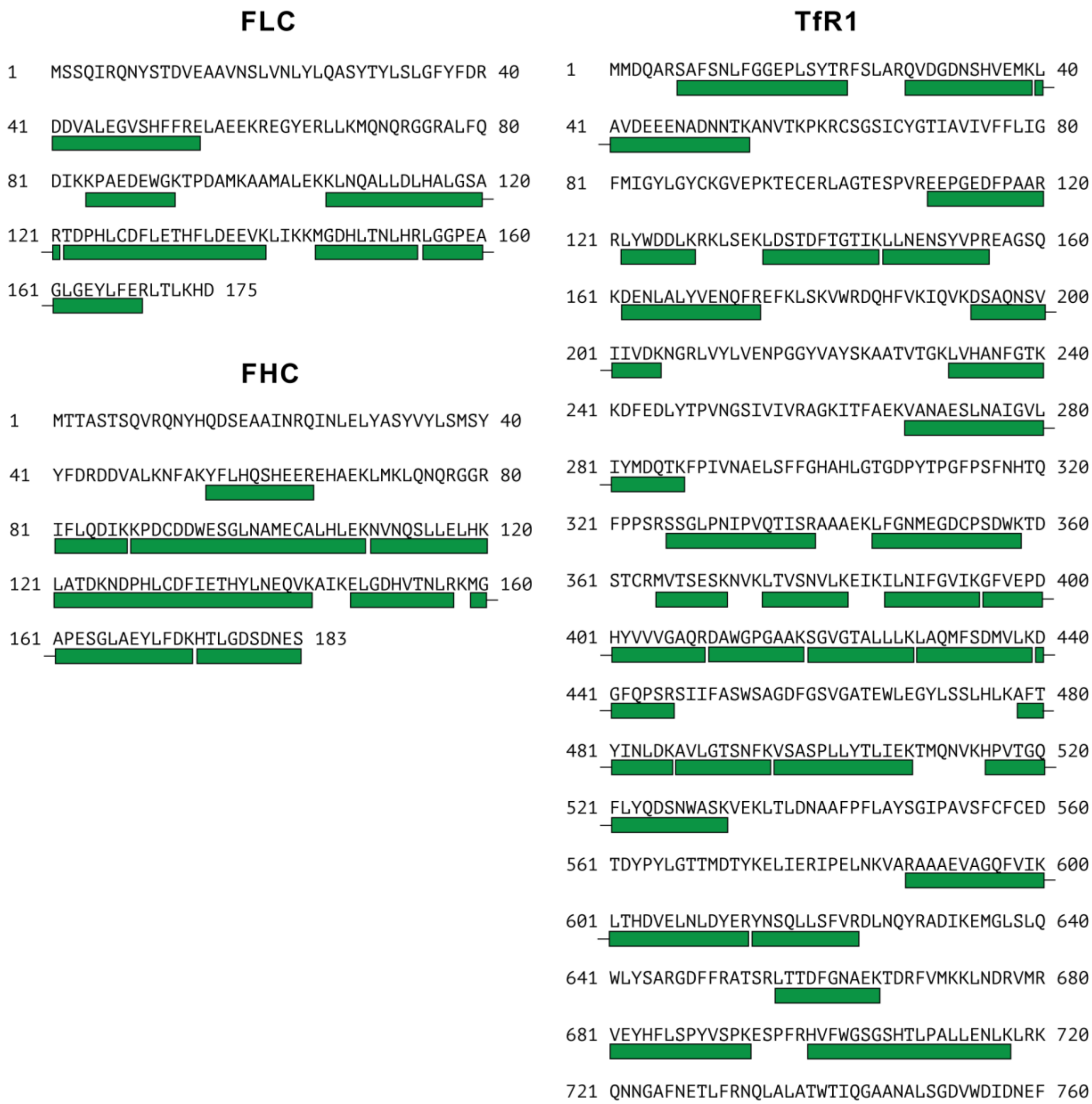

Figure S2. Alignment of peptides identified in purified T. gondii proteome to host FLC, FHC, and TfR1.

Purified RH strain tachyzoites were used for whole proteome purification and sequencing by UHPLC-MS. Non-overlapping or redundant peptides were detected with perfect homology to only human FLC (6 unique peptides), FHC (8 unique peptides), and TfR1 (29 unique peptides). Green bars indicate the coverage of each peptide identified.

Table S1. List of antibodies and reagents used in this study.

Antibody Target / Reagent	Host	Clone / Conjugation	Source	Catalog	Dilution
TfR1	Rabbit IgG	JF0596	Invitrogen	MA5-32500	1:1000 (IFA), 1:500 (FC)
TfR1	Mouse IgG	H68.4	Invitrogen	13-6800	1:500 (IFA, WB)
TfR1	Mouse IgG	OKT-9	Invitrogen	14-0719-82	1:500 (IFA)
TfR1	Human Recomb.	A24	Creative Biolabs	TAB-622CT	1 µg/mL (Blockade)
Tf	Mouse IgG	F2G8H6	Invitrogen	MA5-14690	1:250 (IFA)
Tf-biotin	Goat IgG	Polyclonal	Invitrogen	A80-128B	1:250 (IFA)
FLC	Rabbit IgG	JM10-37	Invitrogen	MA5-32755	1:1000 (IFA)
FHC	Rabbit IgG	Polyclonal	Invitrogen	PA5-27500	1:1000 (IFA)
CTLC	Mouse IgG	2E5	Abnova	H00001213-M05	1:250 (IFA)
GAPDH	Rabbit IgG	Polyclonal	Proteintech	10494-1-AP	1:2000 (WB)
Sag1	Mouse IgG	D61S	Invitrogen	MA5-18628	1:500 (IFA), 1:200 (WB)
Gra5	Mouse IgG	TG 17-113	Biotem	TG 17-113	1:500 (IFA), 1:250 (FC)
Rabbit IgG (H+L)	Goat IgG	Polyclonal, AF 594	Invitrogen	A11012	Variable
Rabbit IgG (H+L)	Goat IgG	Polyclonal, AF 405	Invitrogen	A31556	Variable
Rabbit IgG (H+L)	Goat IgG	Polyclonal, HRP	Invitrogen	G21234	0.5 µg/mL
Mouse IgG (H+L)	Donkey IgG	Polyclonal, AF 488	Invitrogen	A21202	Variable
Mouse IgG (H+L)	Donkey IgG	Polyclonal, HRP	Invitrogen	SA1-100	2 µg/mL
Streptavidin	n/a	AF 594	Invitrogen	S11227	Variable
Dolichos Bifluorus Agglutinin-FITC	*Dolichos bifluorus*	n/a	Invitrogen	L32474	2 µg/mL (IFA)
(Z)-4-hydroxy Tamoxifen	n/a	n/a	Cayman Chemical	14854	2.5 µM
Live/Dead Aqua	n/a	n/a	Invitrogen	L34966	1:1000 (FC)

Abbreviations: TfR1, transferrin receptor 1; Tf, transferrin; FLC, ferritin light chain; FHC, ferritin heavy chain; CTLC, clathrin light chain; GAPDH, glyceraldehyde-3-phosphate dehydrogenase; Sag1, surface antigen 1; Gra5, dense granule protein 5; IgG, immunoglobulin G isotype; H+L, heavy and light chain; FITC, fluorescein isothiocyanate; Recomb, recombinant; AF, Alexa Fluor; IFA, immunofluorescent assay; FC, flow cytometry; WB, western blot.

Variable dilutions were adjusted to match the primary staining reagent.

Table S2. List of primers used in this study.

Target Gene	Application	Primer Sequence (5' to 3')	Primer Dilution (µM)	Product Size (bp)	Reference
Mouse *Ubiquitin-CreER(T2)*	Genotyping	F: 5’-GACGTCACCCGTTCTGTTG-3’	0.5	475 (Cre+)	Jackson Labs
		R: 5’-AGGCAAATTTTGGTGTACGG-3’	0.5	No product (Cre-)	
Mouse *TfR(Flx)*	Genotyping	F: 5’-TTCAGTTCCCAGTGACCACA-3’	0.3	165 (WT)	Jackson Labs
		R: 5’-TCCTTTCTGTGCCCAGTTCT-3’	0.3	310 (Flox)	
T. gondii B1	qPCR (Burdens)	F: 5’-CGTCCGTCGTAATATCAG-3’	0.5	98	38
		R: 5’-GACTTCATGGGACGATATG-3’	0.5		
Mouse *Actin*	qRT-PCR	F: 5’-ACCCACACTGTGCCCTACTA-3’	0.5	112	39
		R: 5’-CACGCTCGGTCAGGATCTTC-3’	0.5		
Mouse *TfR1*	RT-qPCR	F: 5’-TCAAGCCAGATCAATTCTC-3’	0.5	115	40
		R: 5’-AGCCAGTTTCATCTCCACATG-3’	0.5		

Movie Descriptions

Movie S1. Theft of Transferrin Receptor 1 by *T. gondii*

RH-strain *T. gondii* infecting MRC5 fibroblasts for 24 hours was IFA stained for TfR1, Sag1, and DAPI. Z-stack images were taken in 0.05µm intervals. Movie depicts the 3D-projection where TfR1 fluorescence is shown in red, Sag1 fluorescence is shown in green, and DAPI is shown in blue.

Movie S2. Theft of Transferrin by *T. gondii*

RH-strain *T. gondii* infecting MRC5 fibroblasts for 24 hours was IFA stained for TfR1, Tf, and DAPI. Z-stack images were taken in 0.05µm intervals. Movie depicts the 3D-projection where TfR1 fluorescence is shown in red, Tf fluorescence is shown in green, and DAPI is shown in blue.

Movie S3. Inheritance of host TfR1 by *T. gondii* daughter cells during endodyogeny

RH-strain *T. gondii* infecting MRC5 fibroblasts for 24 hours was IFA stained for TfR1, Sag1, and DAPI. Z-stack images were taken in 0.05µm intervals. Movie depicts the 3D-projection where TfR1 fluorescence is shown in red and Sag1 fluorescence is shown in green.

Table S3. Iron-Related Protein Homology Analysis in *Toxoplasma gondii*

Iron Transport & Storage

Protein	Reference Organism	Accession	Target ID	Bit score	E-value	Fitness Score (Sidik et al. 2016)	IFNγ Fitness (Wang et al. 2020)	Reference
DMT1 (Iso 2)	*Mus musculus*	NP_032758.2	TGME49_267270	322	2.00E-95	0.49	-0.38	[1, 2]
NRAMP1	*Mus musculus*	NP_038640.2	TGME49_267270	306	2.00E-90	0.49	-0.38	[3]
VIT	*Plasmodium berghei*	PBANKA_143860	TGME49_266800	255	6.00E-82	-1.22	0.54	[4]
YbtP	*Enterobacteria*	WP_001327262.1	TGME49_269000	209	2.00E-56	-4.96	-0.09	[5]
MntH	*Escherichia coli*	NP_416893.1	TGME49_267270	179	2.00E-47	0.49	-0.38	[6]
DMT1 (Iso 3)	*Mus musculus*	NP_001343881.1	TGME49_267270	162	5.00E-43	0.49	-0.38	[1, 2]
YbtQ	*Enterobacteria*	WP_012131848.1	TGME49_273360	218	3.00E-39	-0.17	-0.24	[5]
EntA	*Escherichia coli*	NP_415128.1	TGME49_217740	103	2.00E-24	-1.89	-0.23	[7, 8]
PchD	*Pseudomonas aeruginosa*	CAA57966.1	TGME49_232580	240	4.00E-20	-4.36	-1.39	[9]
FecE	*Escherichia coli*	AAA97183.1	TGME49_269000	92.8	6.00E-20	-4.96	-0.09	[10]
Irp9	*Enterobacteria*	VTP73725.1	TGME49_202920	235	6.00E-20	-3.74	-0.04	[11]
YbtS	*Enterobacteria*	WP_012132137.1	TGME49_202920	94.7	1.00E-19	-3.74	-0.04	[12]
FhuC	*Escherichia coli*	CAD6022026.1	TGME49_260310	90.1	1.00E-18	0.61	0.46	[13]
YfeB	*Yersinia*	WP_002211843.1	TGME49_273360	208	3.00E-17	-0.17	-0.24	[6]
YbtE	*Enterobacteria*	WP_001088835.1	TGME49_266640	80.9	5.00E-15	0.59	-2.08	[12]
PchA	*Pseudomonas aeruginosa*	CAA57969.1	TGME49_202920	70.9	6.00E-12	-3.74	-0.04	[14]
ZIPCO	*Plasmodium berghei*	PBANKA_0506500	TGME49_261720	59.7	8.00E-09	-4.31	-0.03	[15]
EntC	*Escherichia coli*	NP_415125.1	TGME49_202920	54.7	5.00E-07	-3.74	-0.04	[16]
ZIPCO	*Plasmodium berghei*	PBANKA_0506500	TGME49_225530	52	3.00E-06	-2.94	-0.01	[15]
AlcA	*Bordatella bronchiseptica*	AAB40618.1	No Results					[17]
AlcB	*Bordatella bronchiseptica*	AAB40619.1	No Results					[17]
AlcC	*Bordatella bronchiseptica*	AAB40620.1	No Results					[17]
AlcD	*Bordatella pertussis*	WP_014905923.1	No Results					[18]
AlcE	*Bordatella*	WP_005013753.1	No Results					[19]
AlcS (Bcr)	*Bordatella*	WP_010930940.1	No Results					[20]
Arn1	*Saccharomyces cerevisiae*	NP_011823.1	No Results					[21, 22]
Arn2 (Taf1)	*Saccharomyces cerevisiae*	NP_011816.2	No Results					[22, 23]
Arn3 (Sit1)	*Saccharomyces cerevisiae*	NP_010849.3	No Results					[22, 24]
Arn4 (ENB1)	*Saccharomyces cerevisiae*	NP_014484.1	No Results					[25]
Ceruloplasmin (ferroxidase)	*Homo sapiens*	ACB21047.1	No Results					[26]

CsbX	*Bacillus subtilis*	NP_390654.2	No Results					[27]
DMT1 (Iso 1)	*Mus musculus*	NP_001139633.1	No Results					[1, 2]
EfeB	*Escherichia coli*	NP_415538.1	No Results					[28]
EfeO	*Escherichia coli*	NP_415537.1	No Results					[28]
EfeU	*Escherichia coli*	AKK17243.1	No Results					[28]
EntB	*Escherichia coli*	NP_415127.1	No Results					[29]
EntS	*Escherichia coli*	NP_415123.1	No Results					[30]
Etf-3 (ECH0767)	*Ehrlichia chaffensis*	WP_006011519.1	No Results					[31]
FatA	*Vibrio anguillarum*	CDQ47873.1	No Results					[32]
FatB	*Vibrio anguillarum*	CDQ47872.1	No Results					[33]
FatC	*Vibrio anguillarum*	CDQ47871.1	No Results					[33]
FatD	*Vibrio anguillarum*	P37738.1	No Results					[33]
FauA	*Bordatella bronchiseptica*	WP_062758580.1	No Results					[34]
FecA	*Escherichia coli*	NP_418711.1	No Results					[10, 35]
FecB	*Escherichia coli*	NP_418710.4	No Results					[10, 36]
FecC	*Escherichia coli*	WP_000125187.1	No Results					[10, 36]
FecD	*Escherichia coli*	NP_418708.1	No Results					[10]
FeoA	*Escherichia coli*	NP_417867.1	No Results					[6]
FeoB	*Escherichia coli*	NP_417868.1	No Results					[6]
FeoC	*Escherichia coli*	NP_417869.1	No Results					[6, 37]
Ferroportin	*Mus musculus*	AAF80987.1	No Results					[38, 39]
FET4	*Saccharomyces cerevisiae*	NP_014052.1	No Results					[40]
FeuA	*Bacillus subtilis*	NP_388044.1	No Results					[41]
FeuB	*Bacillus subtilis*	NP_388043.1	No Results					[42]
FeuC	*Bacillus subtilis*	NP_388042.2	No Results					[43]
FhuA	*Escherichia coli*	QIM05368.1	No Results					[44]
FhuB	*Escherichia coli*	CAD6022020.1	No Results					[44]
FhuD	*Escherichia coli*	CAD6017367.1	No Results					[44]
FIT1	*Saccharomyces cerevisiae*	NP_010823.1	No Results					[45]
FIT2	*Saccharomyces cerevisiae*	NP_015027.1	No Results					[45]
FIT3	*Saccharomyces cerevisiae*	NP_015028.3	No Results					[45]
FptA	*Pseudomonas aeruginosa*	NP_252911.1	No Results					[46]
FpvA	*Pseudomonas aeruginosa*	NP_251088.1	No Results					[47]
FRO2	*Arabidopsis thaliana*	CAA70770.1	No Results					[48]
Ftn (Heavy)	*Homo sapiens*	NP_002023.2	No Results					[49]
Ftn (Light)	*Homo sapiens*	NP_000137.2	No Results					[49]
Ftr1	*Saccharomyces cerevisiae*	NP_011072.1	No Results					[50]
FyuA	*Enterobacteria*	WP_000784549.1	No Results					[51]

HemO	*Leptospira interrogans*	AAN51745.1	No Results					[52]
Hepcidin	*Homo sapiens*	AAH20612.1	No Results					[53]
HFE	*Homo sapiens*	AAB82083.1	No Results					[54]
IRT1	*Arabidopsis thaliana*	NP_567590.3	No Results					[55]
IRT2	*Arabidopsis thaliana*	OAO98274.1	No Results					[55]
IucA	*Escherichia coli*	CAA53707.1	No Results					[56]
IucB	*Escherichia coli*	QBQ68952.1	No Results					[56]
IucC	*Escherichia coli*	AAS66995.1	No Results					[56]
IucD	*Escherichia coli*	QBQ68950.1	No Results					[56]
LbpA	*Neisseria meningitidis*	WP_002229702.1	No Results					[57]
LbpB	*Neisseria meningitidis*	WP_196979656.1	No Results					[57]
LbtA	*Legionella pneumophila*	WP_015444551.1	No Results					[58]
LbtB	*Legionella pneumophila*	WP_010947055.1	No Results					[58]
LbtC	*Legionella pneumophila*	WP_010947054.1	No Results					[59]
LbtU	*Legionella pneumophila*	P_010947057.1	No Results					[60]
Lcn2	*Homo sapiens*	XP_047279332.1	No Results					[119]
MexA	*Pseudomonas aeruginosa*	WP_049324879.1	No Results					[61]
MexB	*Pseudomonas aeruginosa*	NP_249117.1	No Results					[61]
OprM	*Pseudomonas aeruginosa*	NP_249118.1	No Results					[61]
PchB	*Pseudomonas aeruginosa*	CAA57968.1	No Results					[9]
PchC	*Pseudomonas aeruginosa*	CAA57967.1	No Results					[62]
PfeA	*Pseudomonas aeruginosa*	NP_251378.1	No Results					[63]
PupB	*Pseudomonas putida*	CAA51995.1	No Results					[64]
PvsC	*Pseudomonas aeruginosa*	PTC36453.1	No Results					[65]
TbpA	*Neisseria meningitidis*	WP_004465694.1	No Results					[66]
TbpB	*Neisseria gonorrhoeae*	STZ83427.1	No Results					[66]
TfR2	*Homo sapiens*	NP_003218.2	No Results					[67]
TfRC	*Homo sapiens*	AAA61153.1	No Results					[68]
TolC	*Escherichia coli*	NP_417507.2	No Results					[69]
Transferrin	*Mus musculus*	NP_598738.1	No Results					[68]
YbtT	*Enterobacteria*	WP_004175339.1	No Results					[70]
YbtU	*Enterobacteria*	WP_012132135.1	No Results					[70]
YbtX	*Enterobacteria*	WP_001286279.1	No Results					[5, 71]
YfeA	*Yersinia*	WP_002211842.1	No Results					[6]
YfeC	*Yersinia*	WP_004706545.1	No Results					[6]
YfeD	*Yersinia*	WP_002211845.1	No Results					[6]
YS1	*Zea mays*	Q9AY27.1	No Results					[6]
YSL1	*Arabidopsis thaliana*	NP_567694.2	No Results					[72]

YSL4	*Arabidopsis thaliana*	NP_198916.2	No Results					[73]
YSL6	*Arabidopsis thaliana*	NP_566806.1	No Results					[73]
ZIP8	*Rattus norvegicus*	AAH89844.1	No Results					[117]
ZIP14	*Mus musculus*	AAH21530.1	No Results					[118]
ZupT	*Escherichia coli*	NP_417512.1	No Results					[6]

Iron-Sulfur Complex Loading

Protein	Reference Organism	Accession	Target ID	Bit score	E-value	Fitness Score (Sidik et al. 2016)	IFNγ Fitness (Wang et al. 2020)	Reference
HscA	*Escherichia coli*	NP_417021.1	TGME49_251780	425	1.00E-137	-5.09	-0.04	[74]
NifS(NFS1)	*Salmonella enterica*	NP_461478.1	TGME49_211090	413	3.00E-137	-3.54	0.02	[74, 75]
SufB	*Plasmodium berghei*	BAL70678.1	TGME49_300650	395	2.00E-131			[76, 77]
HscA	*Escherichia coli*	NP_417021.1	TGME49_311720	399	2.00E-128	-5.5	-0.04	[74]
SufC	*Plasmodium berghei*	SCM17963.1	TGME49_225800	320	3.00E-103	-5.57	-0.04	[76, 77]
SufD	*Plasmodium berghei*	SCO60652.1	TGME49_273445	232	1.00E-60	-5.29	-0.89	[76, 77]
SufS	*Plasmodium berghei*	SCO59130.1	TGME49_216170	206	3.00E-56	-3.95	-0.74	[76-78]
NifU	*Salmonella enterica*	NP_461477.1	TGME49_237560	158	1.00E-47	-5.99	0.17	[74]
Fdx	*Escherichia coli*	NP_417020.1	TGME49_240670	83.2	3.00E-19	-2.45	0.04	[74]
SufA	*Plasmodium berghei*	SCO62101.1	TGME49_297925	87	1.00E-18	-4.01	0.03	[76, 77]
IscA	*Escherichia coli*	NP_417023.1	TGME49_297925	82	8.00E-18	-4.01	0.03	[74]
HscB	*Homo sapiens*	NP_741999.3	TGME49_288685	84.7	1.00E-17	-2.92	-0.05	[74]
SufE	*Plasmodium berghei*	SCM17131.1	TGME49_277010	75.1	1.00E-14	-3.31	-0.11	[76, 77]
CyaY (frataxin)	*Escherichia coli*	NP_418251.1	TGME49_262810	54.7	9.00E-09	-3.34	-0.32	[74]
SufU	*Bacillus subtilis*	QHE14930.1	TGME49_237560	52	1.00E-07	-5.99	0.17	[76, 77]
NfuA	*Escherichia coli*	NP_417873.1	TGME49_221922	46.6	4.00E-05	-0.76	-0.58	[74]
CIA1	*Toxoplasma gondii*	TGME49_313280	N/A	N/A	N/A	-3.95	-0.04	[75]
CIA2	*Toxoplasma gondii*	TGME49_306590	N/A	N/A	N/A	-4.52	-0.03	[75]
Dre2	*Toxoplasma gondii*	TGME49_216900	N/A	N/A	N/A	-2.88	-0.01	[75]
IscU	*Toxoplasma gondii*	TGGT1_327560	N/A	N/A	N/A			[78]
MMS19	*Toxoplasma gondii*	TGME49_222230	N/A	N/A	N/A	-5.93	0.02	[75]
Nar1	*Toxoplasma gondii*	TGME49_232580	N/A	N/A	N/A	-4.36	-1.39	[75]
NBP35	*Toxoplasma gondii*	TGME49_280730	N/A	N/A	N/A	-4.81	-0.01	[75]
Tah18	*Toxoplasma gondii*	TGME49_249320	N/A	N/A	N/A	-3.87	0.01	[75]

Heme Biosynthesis

Protein	Reference Organism	Accession	Target ID	Bit score	E-value	Fitness Score (Sidik et al. 2016)	IFNγ Fitness (Wang et al. 2020)	Reference
ALAS	*Toxoplasma gondii*	TGME49_258690	N/A	N/A	N/A	-4.58	0.49	[79]
PBGS	*Toxoplasma gondii*	TGGT1_253900	N/A	N/A	N/A	-4.41	-0.34	[79]
PBGD	*Toxoplasma gondii*	TGGT1_271420	N/A	N/A	N/A	-3.93	0.06	[79]
UROS	*Toxoplasma gondii*	TGGT1_264200	N/A	N/A	N/A	-4.88	-0.12	[79]
UROD	*Toxoplasma gondii*	TGGT1_289940	N/A	N/A	N/A	-4.6	0.01	[79]
CPOX	*Toxoplasma gondii*	TGGT1_223020	N/A	N/A	N/A	-4.64	-0.02	[79]
PPO	*Toxoplasma gondii*	TGGT1_272490	N/A	N/A	N/A	-3.87	-0.22	[79]
FECH	*Toxoplasma gondii*	TGGT1_258650	N/A	N/A	N/A	-5.69	-0.29	[79]
Tmem14c	*Toxoplasma gondii*	TGGT1_228110	N/A	N/A	N/A	1.29	-0.34	[80]

Regulation

Protein	Reference Organism	Accession	Target ID	Bit score	E-value	Fitness Score (Sidik et al. 2016)	IFNγ Fitness (Wang et al. 2020)	Reference
Aconitase	*Mycobacterium tuberculosis*	O53166.1	TGME49_226730	9421	0.00E+00	-4.73	-0.03	[81]
IRP1	*Homo sapiens*	NP_002188.1	TGME49_226730	1069	0.00E+00	-4.73	-0.03	[82]
IRP2	*Homo sapiens*	AAI17484.1	TGME49_226730	837	0.00E+00	-4.73	-0.03	[83]
Myb2	*Arabidopsis thaliana*	NP_182241.1	TGME49_200385	85.5	4.00E-17	0.98	-0.53	[84, 85]
Myb3	*Arabidopsis thaliana*	NP_564176.2	TGME49_200385	82.8	2.00E-16	0.98	-0.53	[84-86]
AngR	*Vibrio anguillarum*	AAO92378.1	TGME49_232580	186	5.00E-13	1.24	-1.39	[10]
Myb2	*Arabidopsis thaliana*	NP_182241.1	TGME49_275480	52	2.00E-06	-4.24	-0.04	[84]
Myb3	*Arabidopsis thaliana*	NP_564176.2	TGME49_275480	52	2.00E-06	-4.24	-0.04	[84, 86]
Aft1	*Saccaromyces cerevisiae*	NP_011444.1	No Results					[87]
Aft2	*Saccaromyces cerevisiae*	KAF4002108.1	No Results					[88]
AlcR	*Bordatella bronchiseptica*	WP_003814017.1	No Results					[19]
AlgR	*Pseudomonas aeruginosa*	NP_253948.1	No Results					[89]
AlgZ	*Pseudomonas aeruginosa*	NP_253949.1	No Results					[89]
Btr	*Bacillus subtilis*	NP_388045.1	No Results					[10]
DtxR	*Mycobacterium tuberculosis*	NP_217227.1	No Results					[10]
FBXL5	*Homo sapiens*	NP_036293.1	No Results					[82]
FecI	*Escherichia coli*	NP_418713.1	No Results					[10]
FecR	*Escherichia coli*	WP_001068910.1	No Results					[10]
Fer	*Solanum lycopersicum*	NP_001234654.2	No Results					[90]

FIT	*Arabidopsis thaliana*	Q0V7X4.1	No Results					[91]
FIT	*Arabidopsis thaliana*	Q0V7X4.1	No Results					[91]
FIT	*Arabidopsis thaliana*	Q0V7X4.1	No Results					[91]
FpvI(PvdS)	*Pseudomonas aeruginosa*	NP_251077.1	No Results					[10]
FpvR	*Pseudomonas aeruginosa*	NP_251078.1	No Results					[92]
Fur	*Helicobacter pylori*	ABO20835.1	No Results					[93]
Fur	*Escherichia coli*	AHG07399.1	No Results					[10]
IrgB	*Vibrio cholerae*	AOY46299.1	No Results					[10]
Myb1	*Arabidopsis thaliana*	NP_187534.1	No Results					[84]
Myb2	*Arabidopsis thaliana*	NP_182241.1	No Results					[84]
PchA	*Pseudomonas aeruginosa*	AAA25926.1	No Results					[10]
PfeR	*Pseudomonas aeruginosa*	NP_251376.1	No Results					[10]
PfeS	*Pseudomonas aeruginosa*	NP_251377.1	No Results					[10]
PupI	*Pseudomonas putida*	CAA54870.1	No Results					[10]
PupR	*Pseudomonas putida*	CAA54871.1	No Results					[94]
Sre	*Neurospora crassa*	Q1K8E7.1	No Results					[95]
SreA	*Aspergillus nidulans*	AAD25328.1	No Results					[96]
TonB	*Escherichia coli*	NP_415768.1	No Results					[10]
Urbs1	*Ustilago maydis*	AAB05617.1	No Results					[97]
Yap5	*Saccaromyces cerevisiae*	KZV10638.1	No Results					[88]
YbtA	*Yersinia pestis*	WP_000140406.1	No Results					[10]

Redox / Functionality

Protein	Reference Organism	Accession	Target ID	Bit score	E-value	Fitness Score (Sidik et al. 2016)	IFNγ Fitness (Wang et al. 2020)	Reference
COX1	*Homo sapiens*	YP_003024028.1	TGME49_255060	164	7.00E-44	-1.95	-0.11	[98]
Cytochrome C	*Homo sapiens*	NP_061820.1	TGME49_219750	137	5.00E-41	-3.51	-0.72	[99]
eNOS (NOS3) Isoform 1	*Homo sapiens*	NP_000594.2	TGME49_219630	97.1	2.00E-19	-2.78	-0.5	[100]
nNOS (NOS1) Isoform 2	*Homo sapiens*	NP_001191142.1	TGME49_219630	96.7	4.00E-19	-2.78	-0.5	[101]
nNOS (NOS1) Isoform 1	*Homo sapiens*	NP_000611.1	TGME49_219630	96.3	3.00E-19	-2.78	-0.5	[101]
nNOS (NOS1) Isoform 4	*Homo sapiens*	NP_001191147.1	TGME49_219630	96.7	4.00E-19	-2.78	-0.5	[101]
ATPα	*Toxoplasma gondii*	TGME49_204400	N/A	N/A	N/A	-3.84	-0.41	[102-104]
ATPβ	*Toxoplasma gondii*	TGME49_261950	N/A	N/A	N/A	-4.84	-0.04	[102-104]

ATPγ	*Toxoplasma gondii*	TGME49_231910	N/A	N/A	N/A	-3.94	-0.04	[102-104]
ATPδ	*Toxoplasma gondii*	TGME49_226000	N/A	N/A	N/A	-4.57	0.76	[102-104]
ATPε	*Toxoplasma gondii*	TGME49_314820	N/A	N/A	N/A	-3.21	-0.21	[102-104]
ATP OSCP	*Toxoplasma gondii*	TGME49_284540	N/A	N/A	N/A	-3.94	-0.01	[102-104]
ATPc	*Toxoplasma gondii*	TGME49_249720	N/A	N/A	N/A	-2.98	0.1	[102, 103]
ATPb/ICAP2/ASAP-2	*Toxoplasma gondii*	TGME49_231410	N/A	N/A	N/A	-5.37	-0.04	[102-104]
ATPd/ICAP18/ASAP-3	*Toxoplasma gondii*	TGME49_268830	N/A	N/A	N/A	-2.02	0.13	[102-104]
ATPk/ICAP6/ASAP-6	*Toxoplasma gondii*	TGME49_260180	N/A	N/A	N/A	-4.07	0.1	[102-104]
ATPf/ICAP11/ASAP-1(	*Toxoplasma gondii*	TGME49_215610	N/A	N/A	N/A	-3.55	0.16	[102-104]
ATPTG3/ICAP8/ASAP	*Toxoplasma gondii*	TGME49_218940	N/A	N/A	N/A	-3.92	-0.04	[102-104]
ATPTG6/ASAP-4	*Toxoplasma gondii*	TGME49_223040	N/A	N/A	N/A	-4.49	-0.03	[102-104]
ATPTG8/CHCH Domai	*Toxoplasma gondii*	TGME49_258060	N/A	N/A	N/A	-4.07	-0.04	[102, 103]
ATPTG2/ICAP15/ASAI	*Toxoplasma gondii*	TGME49_282180	N/A	N/A	N/A	-2.46	0.18	[102-104]
ATPTG9/ASAP-9/CHC	*Toxoplasma gondii*	TGME49_285510	N/A	N/A	N/A	-1.87	-0.09	[102-104]
ATPa/ASAP-1	*Toxoplasma gondii*	TGME49_310360	N/A	N/A	N/A	-4.49	-0.04	[102-104]
ATPTG15/ICAP9/ASAI	*Toxoplasma gondii*	TGME49_247410	N/A	N/A	N/A	-3.9	-0.63	[102, 104]
IF1	*Toxoplasma gondii*	TGME49_215350	N/A	N/A	N/A	1.82	-1.01	[102-104]
RPS11	*Toxoplasma gondii*	TGME49_226970	N/A	N/A	N/A	-3.75	-0.05	[102]
ApiCox25	*Toxoplasma gondii*	TGGT1_264040	N/A	N/A	N/A	-2.54	0.52	[105]
ApiCox16	*Toxoplasma gondii*	TGGT1_265370	N/A	N/A	N/A	1.56	-0.44	[105]
ApiCox5b	*Toxoplasma gondii*	TGGT1_209260	N/A	N/A	N/A	-3.07	-0.09	[105]
ApiCox18	*Toxoplasma gondii*	TGGT1_221510	N/A	N/A	N/A	-3.28	0.01	[105]
ApiCox23	*Toxoplasma gondii*	TGGT1_262640	N/A	N/A	N/A	-3.49	-0.33	[105]
ApiCox30	*Toxoplasma gondii*	TGGT1_297810	N/A	N/A	N/A	-3.64	-0.17	[105]
ApiCox19	*Toxoplasma gondii*	TGGT1_247770	N/A	N/A	N/A	-2.61	-1.27	[105]
ApiCox35	*Toxoplasma gondii*	TGGT1_229920	N/A	N/A	N/A	-3.84	-0.03	[105]
ApiCox26	*Toxoplasma gondii*	TGGT1_306670	N/A	N/A	N/A	-3.68	-0.18	[105]
ApiCox2a	*Toxoplasma gondii*	TGGT1_226590	N/A	N/A	N/A	-3.8	-0.69	[105]
ApiCox2b	*Toxoplasma gondii*	TGGT1_310470	N/A	N/A	N/A	-4.18	-0.38	[105]
ApiCox24	*Toxoplasma gondii*	TGGT1_286530	N/A	N/A	N/A	-2.82	-0.3	[105]
ApiCox13	*Toxoplasma gondii*	TGGT1_254030	N/A	N/A	N/A	-4.26	-0.04	[105]
ApiCox14	*Toxoplasma gondii*	TGGT1_242840	N/A	N/A	N/A	-3.58	-0.04	[105]
Cox3	*Toxoplasma gondii*	TGVEG_442760	N/A	N/A	N/A			[105]
ATPij/ASAP-11	*Toxoplasma gondii*	TGME49_290030	N/A	N/A	N/A	-3.88	-0.05	[103, 104]
ATPTG17/ASAP-12	*Toxoplasma gondii*	TGME49_310180	N/A	N/A	N/A	-3.4	-0.04	[103, 104]
ATPG10/ASAP-13	*Toxoplasma gondii*	TGME49_214930	N/A	N/A	N/A	-1.37	-0.15	[103, 104]
ATPTG12/ASAP-14	*Toxoplasma gondii*	TGME49_245450	N/A	N/A	N/A	-2.95	-0.08	[103, 104]
ATP8/ASAP-15	*Toxoplasma gondii*	TGME49_208440	N/A	N/A	N/A	-3.54	-0.33	[103, 104]

ATPTG4/ASAP-16	*Toxoplasma gondii*	TGME49_201800	N/A	N/A	N/A	-4.01	0.12	[103, 104]
ATPTG13/ASAP-17	*Toxoplasma gondii*	TGME49_225730	N/A	N/A	N/A	-3.65	-0.25	[103, 104]
ATPTG14/ASAP-18	*Toxoplasma gondii*	TGME49_263080	N/A	N/A	N/A	-3.1	0.06	[103, 104]
ATPTG11/ASAP-19	*Toxoplasma gondii*	TGME49_263990	N/A	N/A	N/A	0.22	-0.13	[103, 104]
ATPTG5/ASAP-20	*Toxoplasma gondii*	TGME49_270360	N/A	N/A	N/A	0.32	-0.24	[103, 104]
ATPTG1	*Toxoplasma gondii*	TGGT1_246540	N/A	N/A	N/A	-4.36	0.04	[103, 104]
ATPTG7	*Toxoplasma gondii*	TGGT1_290710	N/A	N/A	N/A	0.67	-0.41	[103, 104]
ATPTG16	*Toxoplasma gondii*	TGGT1_211060	N/A	N/A	N/A	-3.31	0.26	[103, 104]
Catalase	*Toxoplasma gondii*	TGME49_232250	N/A	N/A	N/A	2.08	-0.25	[106]
Cytochrome p450	*Toxoplasma gondii*	TGGT1_315770	N/A	N/A	N/A	0.80	-1.08	[107]
SOD	*Toxoplasma gondii*	TGME49_316310	N/A	N/A	N/A	-4.42	-0.03	[108]
SOD2	*Toxoplasma gondii*	TGME49_316330	N/A	N/A	N/A	-4.09	-0.04	[109]
SOD3	*Toxoplasma gondii*	TGME49_316190	N/A	N/A	N/A	0.26	-0.09	
Ceruloplasmin	*Homo sapiens*	XP_006713562.1	No Results					[26]
COX4	*Homo sapiens*	NP_001852.1	No Results					[99]
Duodenal cytochrome B reductase	*Mus musculus*	NP_082869.2	No Results					[110]
eNOS (NOS3) Isoform 2	*Homo sapiens*	NP_001153581.1	No Results					[100]
eNOS (NOS3) Isoform 3	*Homo sapiens*	NP_001153582.1	No Results					[100]
eNOS (NOS3) Isoform 4	*Homo sapiens*	NP_001153583.1	No Results					[100]
FET3	*Saccharomyces cerevisiae*	NP_013774.1	No Results					[111]
Fre1	*Saccharomyces cerevisiae*	NP_013315.1	No Results					[22, 112]
Fre2	*Saccharomyces cerevisiae*	CAA82065.1	No Results					[22]
Fre3	*Saccharomyces cerevisiae*	NP_015026.1	No Results					[22]
Fre4	*Saccharomyces cerevisiae*	NP_014458.1	No Results					[22]
Fre5	*Saccharomyces cerevisiae*	NP_015029.1	No Results					[22]
Fre6	*Saccharomyces cerevisiae*	NP_013049.1	No Results					[22]
Hephaestin	*Homo sapiens*	AAK08131.1	No Results					[39, 113, 114]
HMOX-1	*Homo sapiens*	NP_002124.1	No Results					[115]
iNOS Chain A	*Mus musculus*	2ORQ_A	No Results					[100]
iNOS Chain B	*Mus musculus*	1JWK_B	No Results					[100]
Steap1	*Homo sapiens*	NP_036581.1	No Results					[116]
Steap2	*Homo sapiens*	NP_001231873.1	No Results					[116]
Steap3	*Homo sapiens*	NP_878919.2	No Results					[112]
Steap4	*Homo sapiens*	NP_078912.2	No Results					[116]

Table S3. References

1. Fleming, M.D., et al., *Microcytic anaemia mice have a mutation in Nramp2, a candidate iron transporter gene.* Nat Genet, 1997. **16**(4): p. 383-6.
2. Gunshin, H., et al., *Cloning and characterization of a mammalian proton-coupled metal-ion transporter.* Nature, 1997. **388**(6641): p. 482-8.
3. Soe-Lin, S., et al., *Nramp1 promotes efficient macrophage recycling of iron following erythrophagocytosis in vivo.* Proc Natl Acad Sci U S A, 2009. **106**(14): p. 5960-5.
4. Slavic, K., et al., *A vacuolar iron-transporter homologue acts as a detoxifier in Plasmodium.* 2016. **7**: p. 10403.
5. Fetherston, J.D., V.J. Bertolino, and R.D. Perry, *YbtP and YbtQ: two ABC transporters required for iron uptake in Yersinia pestis.* Mol Microbiol, 1999. **32**(2): p. 289-99.
6. Lau, C.K.Y., K.D. Krewulak, and H.J. Vogel, *Bacterial ferrous iron transport: the Feo system.* FEMS Microbiology Reviews, 2016. **40**(2): p. 273-298.
7. Liu, J., K. Duncan, and C.T. Walsh, *Nucleotide sequence of a cluster of Escherichia coli enterobactin biosynthesis genes: identification of entA and purification of its product 2,3-dihydro-2,3-dihydroxybenzoate dehydrogenase.* J Bacteriol, 1989. **171**(2): p. 791-8.
8. Nahlik, M.S., et al., *Nucleotide sequence and transcriptional organization of the Escherichia coli enterobactin biosynthesis cistrons entB and entA.* J Bacteriol, 1989. **171**(2): p. 784-90.
9. Serino, L., et al., *Biosynthesis of pyochelin and dihydroaeruginoic acid requires the iron-regulated pchDCBA operon in Pseudomonas aeruginosa.* J Bacteriol, 1997. **179**(1): p. 248-57.
10. Miethke, M. and M.A. Marahiel, *Siderophore-based iron acquisition and pathogen control.* Microbiol Mol Biol Rev, 2007. **71**(3): p. 413-51.
11. Pelludat, C., D. Brem, and J. Heesemann, *Irp9, encoded by the high-pathogenicity island of Yersinia enterocolitica, is able to convert chorismate into salicylate, the precursor of the siderophore yersiniabactin.* J Bacteriol, 2003. **185**(18): p. 5648-53.
12. Gehring, A.M., et al., *Iron acquisition in plague: modular logic in enzymatic biogenesis of yersiniabactin by Yersinia pestis.* Chem Biol, 1998. **5**(10): p. 573-86.
13. Wooldridge, K.G., J.A. Morrissey, and P.H. Williams, *Transport of ferric-aerobactin into the periplasm and cytoplasm of Escherichia coli K12: role of envelope-associated proteins and effect of endogenous siderophores.* J Gen Microbiol, 1992. **138**(3): p. 597-603.
14. Gaille, C., C. Reimmann, and D. Haas, *Isochorismate synthase (PchA), the first and rate-limiting enzyme in salicylate biosynthesis of Pseudomonas aeruginosa.* J Biol Chem, 2003. **278**(19): p. 16893-8.
15. Sahu, T., et al., *ZIPCO, a putative metal ion transporter, is crucial for Plasmodium liver-stage development.* EMBO Molecular Medicine, 2014. **6**(11): p. 1387-1397.

16. Liu, J., et al., *Overexpression, purification, and characterization of isochorismate synthase (EntC), the first enzyme involved in the biosynthesis of enterobactin from chorismate.* Biochemistry, 1990. **29**(6): p. 1417-25.
17. Kang, H.Y., et al., *Identification and characterization of iron-regulated Bordetella pertussis alcaligin siderophore biosynthesis genes.* J Bacteriol, 1996. **178**(16): p. 4877-84.
18. Funahashi, T., et al., *An iron-regulated gene required for utilization of aerobactin as an exogenous siderophore in Vibrio parahaemolyticus.* Microbiology (Reading), 2003. **149**(Pt 5): p. 1217-1225.
19. Pradel, E., N. Guiso, and C. Locht, *Identification of AlcR, an AraC-type regulator of alcaligin siderophore synthesis in Bordetella bronchiseptica and Bordetella pertussis.* J Bacteriol, 1998. **180**(4): p. 871-80.
20. Brickman, T.J. and S.K. Armstrong, *Bordetella AlcS transporter functions in alcaligin siderophore export and is central to inducer sensing in positive regulation of alcaligin system gene expression.* J Bacteriol, 2005. **187**(11): p. 3650-61.
21. Heymann, P., J.F. Ernst, and G. Winkelmann, *Identification and substrate specificity of a ferrichrome-type siderophore transporter (Arn1p) in Saccharomyces cerevisiae.* FEMS Microbiol Lett, 2000. **186**(2): p. 221-7.
22. Yun, C.W., et al., *Siderophore-iron uptake in saccharomyces cerevisiae. Identification of ferrichrome and fusarinine transporters.* J Biol Chem, 2000. **275**(21): p. 16354-9.
23. Heymann, P., J.F. Ernst, and G. Winkelmann, *Identification of a fungal triacetylfusarinine C siderophore transport gene (TAF1) in Saccharomyces cerevisiae as a member of the major facilitator superfamily.* Biometals, 1999. **12**(4): p. 301-6.
24. Lesuisse, E., M. Simon-Casteras, and P. Labbe, *Siderophore-mediated iron uptake in Saccharomyces cerevisiae: the SIT1 gene encodes a ferrioxamine B permease that belongs to the major facilitator superfamily.* Microbiology (Reading), 1998. **144 (Pt 12)**: p. 3455-3462.
25. Froissard, M., et al., *Trafficking of siderophore transporters in Saccharomyces cerevisiae and intracellular fate of ferrioxamine B conjugates.* Traffic, 2007. **8**(11): p. 1601-16.
26. Van Eden, M.E. and S.D. Aust, *Intact human ceruloplasmin is required for the incorporation of iron into human ferritin.* Arch Biochem Biophys, 2000. **381**(1): p. 119-26.
27. Page, W.J., et al., *The csbX gene of Azotobacter vinelandii encodes an MFS efflux pump required for catecholate siderophore export.* FEMS Microbiol Lett, 2003. **228**(2): p. 211-6.
28. Cao, J., et al., *EfeUOB (YcdNOB) is a tripartite, acid-induced and CpxAR-regulated, low-pH Fe2+ transporter that is cryptic in Escherichia coli K-12 but functional in E. coli O157:H7.* Mol Microbiol, 2007. **65**(4): p. 857-75.
29. Gehring, A.M., K.A. Bradley, and C.T. Walsh, *Enterobactin biosynthesis in Escherichia coli: isochorismate lyase (EntB) is a bifunctional enzyme that is phosphopantetheinylated by EntD and then acylated by EntE using ATP and 2,3-dihydroxybenzoate.* Biochemistry, 1997. **36**(28): p. 8495-503.

30. Holden, V.I., et al., *Iron Acquisition and Siderophore Release by Carbapenem-Resistant Sequence Type 258 Klebsiella pneumoniae.* mSphere, 2018. **3**(2).
31. Yan, Q., et al., *Iron robbery by intracellular pathogen via bacterial effector-induced ferritinophagy.* Proceedings of the National Academy of Sciences of the United States of America, 2021. **118**(23).
32. Köster, W.L., et al., *Molecular characterization of the iron transport system mediated by the pJM1 plasmid in Vibrio anguillarum 775.* J Biol Chem, 1991. **266**(35): p. 23829-33.
33. Naka, H., C.S. López, and J.H. Crosa, *Role of the pJM1 plasmid-encoded transport proteins FatB, C and D in ferric anguibactin uptake in the fish pathogen Vibrio anguillarum.* Environ Microbiol Rep, 2010. **2**(1): p. 104-111.
34. Brickman, T.J. and S.K. Armstrong, *Essential role of the iron-regulated outer membrane receptor FauA in alcaligin siderophore-mediated iron uptake in Bordetella species.* J Bacteriol, 1999. **181**(19): p. 5958-66.
35. Chakraborty, R., E. Storey, and D. van der Helm, *Molecular mechanism of ferricsiderophore passage through the outer membrane receptor proteins of Escherichia coli.* Biometals, 2007. **20**(3-4): p. 263-74.
36. Braun, V. and F. Endriss, *Energy-coupled outer membrane transport proteins and regulatory proteins.* Biometals, 2007. **20**(3-4): p. 219-31.
37. Smith, A.T., et al., *The FeoC [4Fe-4S] Cluster Is Redox-Active and Rapidly Oxygen-Sensitive.* Biochemistry, 2019. **58**(49): p. 4935-4949.
38. Abboud, S. and D.J. Haile, *A novel mammalian iron-regulated protein involved in intracellular iron metabolism.* J Biol Chem, 2000. **275**(26): p. 19906-12.
39. Han, O. and E.Y. Kim, *Colocalization of ferroportin-1 with hephaestin on the basolateral membrane of human intestinal absorptive cells.* J Cell Biochem, 2007. **101**(4): p. 1000-10.
40. Dix, D., et al., *Characterization of the FET4 protein of yeast. Evidence for a direct role in the transport of iron.* J Biol Chem, 1997. **272**(18): p. 11770-7.
41. Peuckert, F., et al., *The siderophore binding protein FeuA shows limited promiscuity toward exogenous triscatecholates.* Chem Biol, 2011. **18**(7): p. 907-19.
42. Ichihara, S. and S. Mizushima, *Identification of an outer membrane protein responsible for the binding of the Fe-enterochelin complex to Escherichia coli cells.* J Biochem, 1978. **83**(1): p. 137-40.
43. Urzúa, L.S., et al., *Identification and characterization of an iron ABC transporter operon in Gluconacetobacter diazotrophicus Pal 5.* Arch Microbiol, 2013. **195**(6): p. 431-8.
44. Fecker, L. and V. Braun, *Cloning and expression of the fhu genes involved in iron(III)-hydroxamate uptake by Escherichia coli.* J Bacteriol, 1983. **156**(3): p. 1301-14.
45. Protchenko, O., et al., *Three cell wall mannoproteins facilitate the uptake of iron in Saccharomyces cerevisiae.* J Biol Chem, 2001. **276**(52): p. 49244-50.
46. Hoegy, F., et al., *Binding of iron-free siderophore, a common feature of siderophore outer membrane transporters of Escherichia coli and Pseudomonas aeruginosa.* J Biol Chem, 2005. **280**(21): p. 20222-30.

47. Nader, M., et al., *Mechanism of ferripyoverdine uptake by Pseudomonas aeruginosa outer membrane transporter FpvA: no diffusion channel formed at any time during ferrisiderophore uptake.* Biochemistry, 2011. **50**(13): p. 2530-40.
48. Kim, S.A., et al., *The iron deficiency response in Arabidopsis thaliana requires the phosphorylated transcription factor URI.* Proc Natl Acad Sci U S A, 2019. **116**(50): p. 24933-24942.
49. Harrison, P.M. and P. Arosio, *The ferritins: molecular properties, iron storage function and cellular regulation.* Biochimica et Biophysica Acta (BBA) - Bioenergetics, 1996. **1275**(3): p. 161-203.
50. Askwith, C. and J. Kaplan, *An oxidase-permease-based iron transport system in Schizosaccharomyces pombe and its expression in Saccharomyces cerevisiae.* J Biol Chem, 1997. **272**(1): p. 401-5.
51. Haag, H., et al., *Purification of yersiniabactin: a siderophore and possible virulence factor of Yersinia enterocolitica.* J Gen Microbiol, 1993. **139**(9): p. 2159-65.
52. Murray, G.L., et al., *Leptospira interrogans requires a functional heme oxygenase to scavenge iron from hemoglobin.* Microbes Infect, 2008. **10**(7): p. 791-7.
53. Pigeon, C., et al., *A new mouse liver-specific gene, encoding a protein homologous to human antimicrobial peptide hepcidin, is overexpressed during iron overload.* J Biol Chem, 2001. **276**(11): p. 7811-9.
54. Barton, J.C., C.Q. Edwards, and R.T. Acton, *HFE gene: Structure, function, mutations, and associated iron abnormalities.* Gene, 2015. **574**(2): p. 179-92.
55. Vert, G., et al., *IRT1, an Arabidopsis transporter essential for iron uptake from the soil and for plant growth.* Plant Cell, 2002. **14**(6): p. 1223-33.
56. de Lorenzo, V., et al., *Aerobactin biosynthesis and transport genes of plasmid ColV-K30 in Escherichia coli K-12.* J Bacteriol, 1986. **165**(2): p. 570-8.
57. Bonnah, R.A. and A.B. Schryvers, *Preparation and characterization of Neisseria meningitidis mutants deficient in production of the human lactoferrin-binding proteins LbpA and LbpB.* J Bacteriol, 1998. **180**(12): p. 3080-90.
58. Allard, K.A., et al., *Purification of Legiobactin and importance of this siderophore in lung infection by Legionella pneumophila.* Infect Immun, 2009. **77**(7): p. 2887-95.
59. Chatfield, C.H., et al., *The major facilitator superfamily-type protein LbtC promotes the utilization of the legiobactin siderophore by Legionella pneumophila.* Microbiology (Reading), 2012. **158**(Pt 3): p. 721-735.
60. Chatfield, C.H., et al., *Legionella pneumophila LbtU acts as a novel, TonB-independent receptor for the legiobactin siderophore.* J Bacteriol, 2011. **193**(7): p. 1563-75.
61. Poole, K., et al., *Multiple antibiotic resistance in Pseudomonas aeruginosa: evidence for involvement of an efflux operon.* J Bacteriol, 1993. **175**(22): p. 7363-72.
62. Serino, L., et al., *Structural genes for salicylate biosynthesis from chorismate in Pseudomonas aeruginosa.* Mol Gen Genet, 1995. **249**(2): p. 217-28.
63. Gasser, V., et al., *Catechol siderophores repress the pyochelin pathway and activate the enterobactin pathway in Pseudomonas aeruginosa: an opportunity*

for siderophore-antibiotic conjugates development. Environ Microbiol, 2016. **18**(3): p. 819-32.
64. Koster, M., et al., *Role for the outer membrane ferric siderophore receptor PupB in signal transduction across the bacterial cell envelope.* Embo j, 1994. **13**(12): p. 2805-13.
65. Tanabe, T., et al., *Identification and characterization of genes required for biosynthesis and transport of the siderophore vibrioferrin in Vibrio parahaemolyticus.* J Bacteriol, 2003. **185**(23): p. 6938-49.
66. Powell, N.B., et al., *Differential binding of apo and holo human transferrin to meningococci and co-localisation of the transferrin-binding proteins (TbpA and TbpB).* J Med Microbiol, 1998. **47**(3): p. 257-64.
67. Chen, J., et al., *Transferrin-directed internalization and cycling of transferrin receptor 2.* Traffic, 2009. **10**(10): p. 1488-501.
68. Enns, C.A., E.A. Rutledge, and A.M. Williams, *The transferrin receptor*, in *Biomembranes: A Multi-Volume Treatise*, A.G. Lee, Editor. 1996, JAIa. p. 255-287.
69. Bleuel, C., et al., *TolC is involved in enterobactin efflux across the outer membrane of Escherichia coli.* J Bacteriol, 2005. **187**(19): p. 6701-7.
70. Geoffroy, V.A., J.D. Fetherston, and R.D. Perry, *Yersinia pestis YbtU and YbtT are involved in synthesis of the siderophore yersiniabactin but have different effects on regulation.* Infect Immun, 2000. **68**(8): p. 4452-61.
71. Bobrov, A.G., et al., *Zinc transporters YbtX and ZnuABC are required for the virulence of Yersinia pestis in bubonic and pneumonic plague in mice.* Metallomics, 2017. **9**(6): p. 757-772.
72. Waters, B.M., et al., *Mutations in Arabidopsis yellow stripe-like1 and yellow stripe-like3 reveal their roles in metal ion homeostasis and loading of metal ions in seeds.* Plant Physiol, 2006. **141**(4): p. 1446-58.
73. Divol, F., et al., *The Arabidopsis YELLOW STRIPE LIKE4 and 6 transporters control iron release from the chloroplast.* Plant Cell, 2013. **25**(3): p. 1040-55.
74. Lill, R., *Function and biogenesis of iron-sulphur proteins.* Nature, 2009. **460**(7257): p. 831-8.
75. Aw, Y.T.V., et al., *A key cytosolic iron-sulfur cluster synthesis protein localizes to the mitochondrion of Toxoplasma gondii.* Mol Microbiol, 2021. **115**(5): p. 968-985.
76. Charan, M., et al., *Sulfur Mobilization for Fe-S Cluster Assembly by the Essential SUF Pathway in the Plasmodium falciparum Apicoplast and Its Inhibition.* Antimicrobial Agents and Chemotherapy, 2014. **58**(6): p. 3389-3398.
77. Ellis, K.E., et al., *Nifs and Sufs in malaria.* Mol Microbiol, 2001. **41**(5): p. 973-81.
78. Pamukcu, S., et al., *Differential contribution of two organelles of endosymbiotic origin to iron-sulfur cluster synthesis and overall fitness in Toxoplasma.* PLoS Pathog, 2021. **17**(11): p. e1010096.
79. Bergmann, A., et al., *Toxoplasma gondii requires its plant-like heme biosynthesis pathway for infection.* PLoS Pathog, 2020. **16**(5): p. e1008499.
80. Harding, C.R., et al., *Genetic screens reveal a central role for heme metabolism in artemisinin susceptibility.* Nature Communications, 2020. **11**(1): p. 4813.

81. Banerjee, S., et al., *Iron-dependent RNA-binding activity of Mycobacterium tuberculosis aconitase.* J Bacteriol, 2007. **189**(11): p. 4046-52.
82. Johnson, N.B., et al., *A synergistic role of IRP1 and FBXL5 proteins in coordinating iron metabolism during cell proliferation.* J Biol Chem, 2017. **292**(38): p. 15976-15989.
83. Hanson, E.S., M.L. Rawlins, and E.A. Leibold, *Oxygen and iron regulation of iron regulatory protein 2.* J Biol Chem, 2003. **278**(41): p. 40337-42.
84. Hsu, H.M., et al., *Transcriptional regulation of an iron-inducible gene by differential and alternate promoter entries of multiple Myb proteins in the protozoan parasite Trichomonas vaginalis.* Eukaryot Cell, 2009. **8**(3): p. 362-72.
85. Waldman, B.S., et al., *Identification of a Master Regulator of Differentiation in Toxoplasma.* Cell, 2020. **180**(2): p. 359-372.e16.
86. Hsu, H.M., et al., *Regulation of nuclear translocation of the Myb1 transcription factor by TvCyclophilin 1 in the protozoan parasite Trichomonas vaginalis.* J Biol Chem, 2014. **289**(27): p. 19120-36.
87. Yamaguchi-Iwai, Y., A. Dancis, and R.D. Klausner, *AFT1: a mediator of iron regulated transcriptional control in Saccharomyces cerevisiae.* Embo j, 1995. **14**(6): p. 1231-9.
88. Martínez-Pastor, M.T., A. Perea-García, and S. Puig, *Mechanisms of iron sensing and regulation in the yeast Saccharomyces cerevisiae.* World J Microbiol Biotechnol, 2017. **33**(4): p. 75.
89. Little, A.S., et al., *Pseudomonas aeruginosa AlgR Phosphorylation Status Differentially Regulates Pyocyanin and Pyoverdine Production.* mBio, 2018. **9**(1).
90. Brumbarova, T. and P. Bauer, *Iron-mediated control of the basic helix-loop-helix protein FER, a regulator of iron uptake in tomato.* Plant Physiol, 2005. **137**(3): p. 1018-26.
91. Yuan, Y., et al., *FIT interacts with AtbHLH38 and AtbHLH39 in regulating iron uptake gene expression for iron homeostasis in Arabidopsis.* Cell Res, 2008. **18**(3): p. 385-97.
92. Beare, P.A., et al., *Siderophore-mediated cell signalling in Pseudomonas aeruginosa: divergent pathways regulate virulence factor production and siderophore receptor synthesis.* Mol Microbiol, 2003. **47**(1): p. 195-207.
93. Bagg, A. and J.B. Neilands, *Ferric uptake regulation protein acts as a repressor, employing iron (II) as a cofactor to bind the operator of an iron transport operon in Escherichia coli.* Biochemistry, 1987. **26**(17): p. 5471-7.
94. Koster, M., et al., *Identification and characterization of the pupB gene encoding an inducible ferric-pseudobactin receptor of Pseudomonas putida WCS358.* Mol Microbiol, 1993. **8**(3): p. 591-601.
95. Zhou, L.W., H. Haas, and G.A. Marzluf, *Isolation and characterization of a new gene, sre, which encodes a GATA-type regulatory protein that controls iron transport in Neurospora crassa.* Mol Gen Genet, 1998. **259**(5): p. 532-40.
96. Haas, H., et al., *The Aspergillus nidulans GATA factor SREA is involved in regulation of siderophore biosynthesis and control of iron uptake.* J Biol Chem, 1999. **274**(8): p. 4613-9.

97. Voisard, C., et al., *urbs1, a gene regulating siderophore biosynthesis in Ustilago maydis, encodes a protein similar to the erythroid transcription factor GATA-1.* Mol Cell Biol, 1993. **13**(11): p. 7091-100.
98. Tran, N.T., et al., *Antiplatelet activity of deferiprone through cyclooxygenase-1 inhibition.* Platelets, 2020. **31**(4): p. 505-512.
99. Semenza, G.L., *Hypoxia-inducible factor 1: regulator of mitochondrial metabolism and mediator of ischemic preconditioning.* Biochim Biophys Acta, 2011. **1813**(7): p. 1263-8.
100. Alderton, W.K., C.E. Cooper, and R.G. Knowles, *Nitric oxide synthases: structure, function and inhibition.* Biochem J, 2001. **357**(Pt 3): p. 593-615.
101. Morishima, Y., et al., *Heme-dependent activation of neuronal nitric oxide synthase by cytosol is due to an Hsp70-dependent, thioredoxin-mediated thiol-disulfide interchange in the heme/substrate binding cleft.* Biochemistry, 2011. **50**(33): p. 7146-56.
102. Huet, D., et al., *Identification of cryptic subunits from an apicomplexan ATP synthase.* Elife, 2018. **7**.
103. Mühleip, A., et al., *ATP synthase hexamer assemblies shape cristae of Toxoplasma mitochondria.* Nat Commun, 2021. **12**(1): p. 120.
104. Salunke, R., et al., *Highly diverged novel subunit composition of apicomplexan F-type ATP synthase identified from Toxoplasma gondii.* PLoS Biol, 2018. **16**(7): p. e2006128.
105. Seidi, A., et al., *Elucidating the mitochondrial proteome of Toxoplasma gondii reveals the presence of a divergent cytochrome c oxidase.* Elife, 2018. **7**.
106. Sibley, L.D., R. Lawson, and E. Weidner, *Superoxide dismutase and catalase in Toxoplasma gondii.* Mol Biochem Parasitol, 1986. **19**(1): p. 83-7.
107. Zhang, X., et al., *Functional characterization of a unique cytochrome P450 in Toxoplasma gondii.* Oncotarget, 2017. **8**(70): p. 115079-115088.
108. Odberg-Ferragut, C., et al., *Molecular cloning, expression analysis and iron metal cofactor characterisation of a superoxide dismutase from Toxoplasma gondii.* Mol Biochem Parasitol, 2000. **106**(1): p. 121-9.
109. Brydges, S.D. and V.B. Carruthers, *Mutation of an unusual mitochondrial targeting sequence of SODB2 produces multiple targeting fates in Toxoplasma gondii.* Journal of Cell Science, 2003. **116**(22): p. 4675.
110. McKie, A.T., et al., *An iron-regulated ferric reductase associated with the absorption of dietary iron.* Science, 2001. **291**(5509): p. 1755-9.
111. De Silva, D.M., et al., *The FET3 gene product required for high affinity iron transport in yeast is a cell surface ferroxidase.* J Biol Chem, 1995. **270**(3): p. 1098-101.
112. Ohgami, R.S., et al., *Identification of a ferrireductase required for efficient transferrin-dependent iron uptake in erythroid cells.* Nat Genet, 2005. **37**(11): p. 1264-9.
113. Levy, J.E., L.K. Montross, and N.C. Andrews, *Genes that modify the hemochromatosis phenotype in mice.* J Clin Invest, 2000. **105**(9): p. 1209-16.
114. Dupic, F., et al., *Duodenal mRNA expression of iron related genes in response to iron loading and iron deficiency in four strains of mice.* Gut, 2002. **51**(5): p. 648-53.

115. Immenschuh, S., E. Baumgart-Vogt, and S. Mueller, *Heme oxygenase-1 and iron in liver inflammation: a complex alliance.* Curr Drug Targets, 2010. **11**(12): p. 1541-50.
116. Ohgami, R.S., et al., *The Steap proteins are metalloreductases.* Blood, 2006. **108**(4): p. 1388-94.
117. Wang, C.Y., et al., *ZIP8 is an iron and zinc transporter whose cell-surface expression is up-regulated by cellular iron loading.* J Biol Chem, 2012. **287**(41): p. 34032-43.
118. Pinilla-Tenas, J.J., et al., *Zip14 is a complex broad-scope metal-ion transporter whose functional properties support roles in the cellular uptake of zinc and nontransferrin-bound iron.* Am J Physiol Cell Physiol, 2011. **301**(4): p. C862-71.
119. Goetz, D.H., et al., *The neutrophil lipocalin NGAL is a bacteriostatic agent that interferes with siderophore-mediated iron acquisition.* Mol Cell, 2002. **10**(5): p. 1033-43.
